# Purinoreceptors and ectonucleotidases control ATP-induced calcium waveforms and calcium-dependent responses in microglia: Roles of P2 receptors and CD39 in ATP-stimulated microglia

**DOI:** 10.3389/fphys.2022.1037417

**Published:** 2023-01-09

**Authors:** Byeong J. Chun, Surya P. Aryal, Peter Varughese, Bin Sun, Joshua A. Bruno, Chris I. Richards, Adam D. Bachstetter, Peter M. Kekenes-Huskey

**Affiliations:** ^1^ Department of Cell and Molecular Physiology, Loyola University Chicago, Chicago, IL, United States; ^2^ Department of Chemistry, University of Kentucky, Lexington, KY, United States; ^3^ Department of Physics, Loyola University Chicago, Chicago, IL, United States; ^4^ College of Medicine, University of Kentucky, Lexington, KY, United States

**Keywords:** cells-monocytes/macrophages, processes-cell activation, processes-chemotaxis, processes-inflammation, processes-signal transduction, calcium

## Abstract

Adenosine triphosphate (ATP) and its metabolites drive microglia migration and cytokine production by activating P2X- and P2Y- class purinergic receptors. Purinergic receptor activation gives rise to diverse intracellular calcium (Ca2+ signals, or waveforms, that differ in amplitude, duration, and frequency. Whether and how these characteristics of diverse waveforms influence microglia function is not well-established. We developed a computational model trained with data from published primary murine microglia studies. We simulate how purinoreceptors influence Ca2+ signaling and migration, as well as, how purinoreceptor expression modifies these processes. Our simulation confirmed that P2 receptors encode the amplitude and duration of the ATP-induced Ca2+ waveforms. Our simulations also implicate CD39, an ectonucleotidase that rapidly degrades ATP, as a regulator of purinergic receptor-induced Ca2+ responses. Namely, it was necessary to account for CD39 metabolism of ATP to align the model’s predicted purinoreceptor responses with published experimental data. In addition, our modeling results indicate that small Ca2+ transients accompany migration, while large and sustained transients are needed for cytokine responses. Lastly, as a proof-of-principal, we predict Ca2+ transients and cell membrane displacements in a BV2 microglia cell line using published P2 receptor mRNA data to illustrate how our computer model may be extrapolated to other microglia subtypes. These findings provide important insights into how differences in purinergic receptor expression influence microglial responses to ATP.

## 1 Introduction

Microglia are the macrophages of the central nervous system (CNS). They contribute to homeostatic and innate immune responses when subject to a spectrum of molecular stimuli, including those associated with infection and cellular damage (Nayak et al., 2014). Microglia respond to these stimuli by migrating, undergoing changes in protein expression, secreting cytokines and chemokines to engage the adaptive immune response, and phagocytosing foreign bodies (Nayak et al., 2014). Many details of these complex signaling pathways controlling microglial responses to such cues are beginning to emerge, including those mediated by ATP and its derivatives (Kigerl et al., 2014).

Extracellular ATP invokes Ca^2+^ fluctuations in many cells. These fluctuations are frequently and interchangeably referred to as ‘transients’ or ‘waveforms’ in the literature. In other cell types, the waveform of an induced Ca^2+^ signal, that is, its duration, amplitude, and frequency, has been shown to selectively control intracellular processes including phosphorylation, gene transcription and mechanical responses (Clapham, 2007). In microglia, Ca^2+^ waveforms are known to trigger cytokine and migration responses (Hide et al., 2000; Ohsawa et al., 2007; Ikeda et al., 2013), as well as a broad set of microglial signaling pathways (Kettenmann et al., 2011; Noda et al., 2012). Despite these observations, it has yet to be determined if the dynamic properties of Ca^2+^ waveforms in microglia exhibit similar selective control of physiological functions as observed in other cell types.

ATP-dependent responses in microglia are mediated by purinergic (P2) receptors. P2 receptors are broadly categorized into two classes: ionotropic (*P*2*X*) and metabotropic (*P*2*Y*) receptors. Ionotropic *P*2*X* receptors are non-selective cation channels widely expressed in cells throughout the CNS including microglia. Of these, P2X7 and P2X4 tend to be the most highly-expressed *P*2*X*-class receptors in microglia (Kettenmann et al., 2011; Asatryan et al., 2018). In our previous work (Chun et al., 2019), we developed a computational model demonstrating that *P*2*X* activation promotes the production of a pro-inflammatory cytokine, TNF*α*. However, microglia also express *P*2*Y* receptors that comprise G protein coupled receptors (GPCR) that can mediate pathways including ER Ca^2+^ release (Kettenmann et al., 2011; Noda et al., 2012). There are several prominent *P*2*Y* receptors present in microglia that respond to diverse nucleotides including ATP, adenosine diphosphate (ADP), and uridine triphosphate (UTP). Among these are *P*2*Y*2 (ATP/UTP), *P*2*Y*6 (UDP), *P*2*Y*12, and *P*2*Y*13 (ATP/ADP) (Kettenmann et al., 2011; Hickman et al., 2013). In principle, both classes of P2 receptors contribute to ATP-mediated responses in microglia, but their simultaneous contributions have yet to be determined in quantitative detail.

Microglia exhibit membrane displacements, such as extensions and retractions of plasma membrane, in response to ATP and can migrate toward sources of ATP (Orr et al., 2009). Ca^2+^ transients that coincide with directed migration have been observed in microglia (Ohsawa et al., 2004; Ohsawa et al., 2007; Ikeda et al., 2013; Franco-Bocanegra et al., 2019). Given the diverse Ca^2+^ waveforms induced by P2 activation, there is an intriguing possibility that microglia adopt unique cell responses to different waveforms that could select for migration *versus* inflammatory behaviors. However, it remains to be determined if variable Ca^2+^ waveforms are just a consequence of ATP stimulation or if they selectively influence cell functions.

**FIGURE 1:**
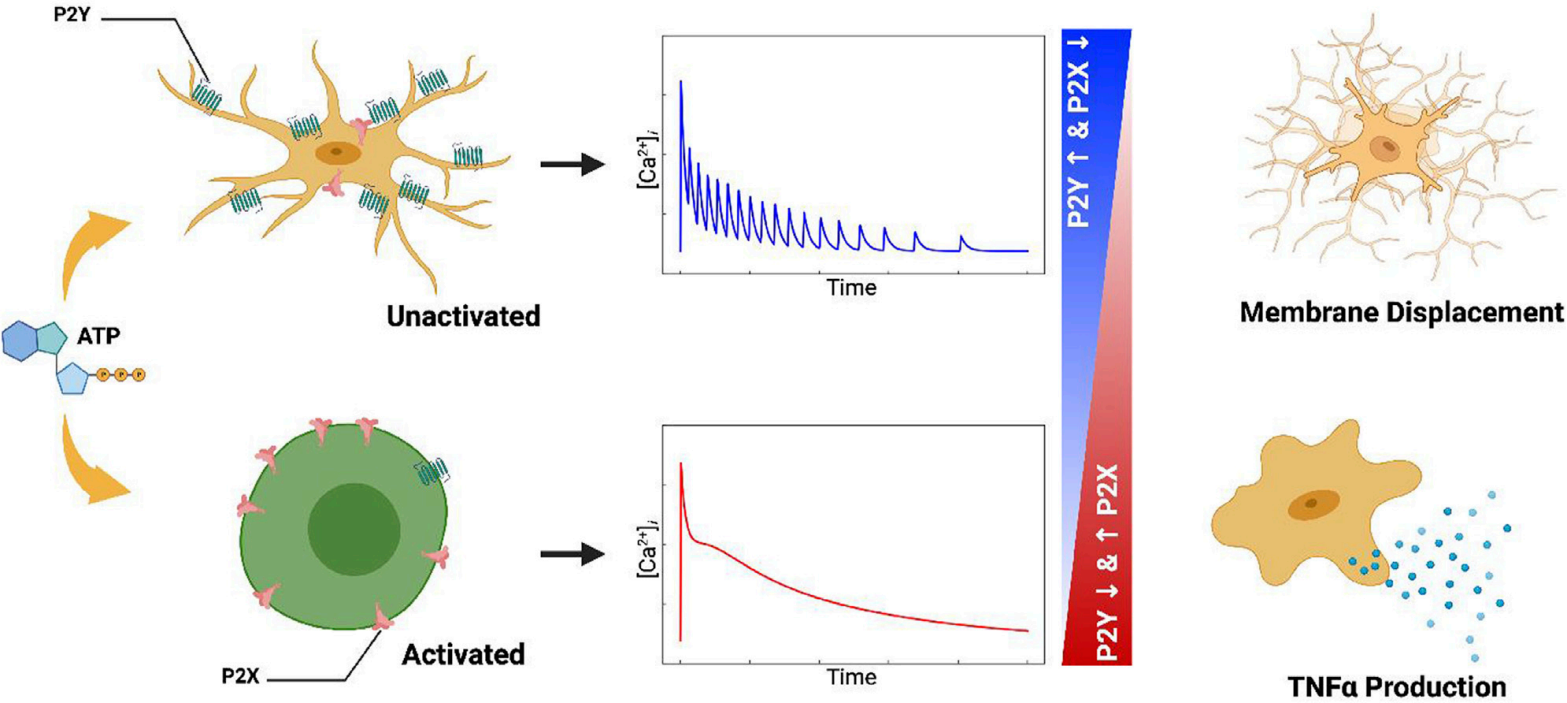
Table of Contents Graphic. Schematic of the computational model for ATP triggered *P*2*Y* and *P*2*X* receptors expressed in microglia (left column). The computational model predicts cellular responses including intracellular Ca^2+^ signals (middle column), migration, and cytokine production (right column) in response to ATP.

Ca^2+^ waveforms and the capacity for cell migration in response to ATP are dependent on P2 expression and activity (Ferreira and Schlichter, 2013; Langfelder et al., 2015). P2 subtype expression can vary considerably among microglial subpopulations and activation states (Bianco et al., 2005; Crain and Watters, 2015; He et al., 2018). As an example, resting, *in vivo* microglia are characterized by having high *P*2*Y*12 expression and comparatively low expression of P2X4 and P2X7 (Crain et al., 2009; Kettenmann et al., 2011; Gomez Morillas et al., 2021), whereas classically activated microglia upregulate P2X4 and downregulate *P*2*Y*12 (Gomez Morillas et al., 2021). This motivated our hypothesis that *P*2*X* and P2Y co-expression in microglia subpopulations enable the cells to encode unique Ca^2+^ waveforms that prime membrane displacement *versus* inflammatory responses (Figure 1). This intuitive hypothesis is complicated by observations that ectonucleotidases, which degrade nucleotides like ATP, help determine the pool of ATP available (Sandefur et al., 2017; Rahmaninejad et al., 2020) to stimulate purinoreceptors.

To investigate this hypothesis, we expanded our model of P2X4/P2X7 activation in microglia (Chun et al., 2019) to include contributions from *P*2*Y*-class receptors. The extended model includes G-protein mediated Ca^2+^ signaling and the activation of pathways implicated in microglia migration. This approach complements prior computational studies of Ca^2+^ responses induced by *P*2*X* receptors (Khadra et al., 2012; Chun et al., 2019) and metabotropic receptors that promote intracellular Ca^2+^ release (Cuthbertson and Chay, 1991; Skupin et al., 2008; Skupin et al., 2010). With this model, we specifically examined how *P*2*X*- and *P*2*Y*-class purinoreceptors encode ATP-triggered Ca^2+^ waveforms in microglia, how these waveforms are modulated by the ectonucleotidase CD39, and how these waveforms influence migration and cytokine responses.

## 2 Materials and methods

### 2.1 The computational model

**FIGURE 2:**
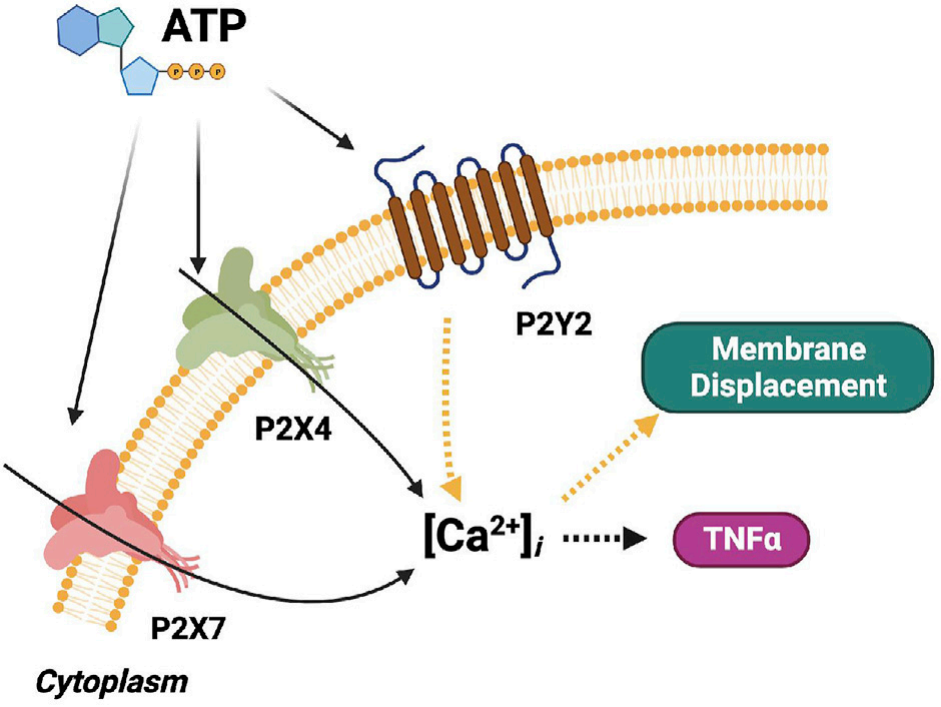
Schematic of the computational microglia model, which simulates the ATP-dependent activation of *P*2*X*- and *P*2*Y*-class receptors. The simulations also account for increases in intracellular Ca^2+^, cell membrane displacement, and TNF*α* cytokine production following P2 receptor activation. The pathways modeled in (Chun et al., 2019) for *P*2*X* signaling are shown in black. The pathways modeled for *P*2*Y* signaling are shown in yellow. Pathways added in this study are shown in orange. The dashed arrows denote multi-step cascades.

We extended the computational model of P2X4-and P2X7-mediated Ca^2+^ signals and TNF*α* production described in Chun et al. (2019) to include *P*2*Y*-dependent contributions to membrane displacement and cytokine responses (see Figure 2). The original model consisted of differential-equation based descriptions of P2X4 and P2X7 gating (Chun et al., 2019), Ca^2+^ influx *via* *P*2*X* channels, and the production of TNF*α*. The aforementioned model has been extended with the addition of pathways including PI3K activation and phosphorylation of Akt to provide a quantitative measure of microglial migration with respect to ATP (Ohsawa et al., 2007). Equations and parameters are provided in the supplement.

The P2X4 and *P*2*X*7 models used in this study were implemented as described in Chun et al. (2019), which were originally derived from published models (Yan et al., 2010; Khadra et al., 2012; Toulme and Khakh, 2012). To describe the dynamics of cytosolic Ca^2+^, we implemented and validated Ca^2+^ uptake and release from the ER by SERCA and *IP*_3_ *R*, respectively. Basal Ca^2+^ levels are restored by the SERCA pump and the NCX as described in (Chun et al., 2019).

For the metabotropic receptor contributions, we assumed that *P*2*Y*2 activation promotes the disassembly of the *G*_*αq*_ subunit, after which PLC-*β* is activated to promote the generation of *IP*_3_ and DAG from *PIP*_2_ (Cuthbertson and Chay, 1991; Kettenmann et al., 2011). We described this process using a mathematical model introduced by Cutherbertson and Chay (Cuthbertson and Chay, 1991) for *IP*_3_-mediated Ca^2+^ oscillations in oocytes. The model includes receptors for *G*_*αq*_ protein activation, *G*_*αq*_-dependent PLC-*β* activation, PLC-*β*-dependent *IP*_3_ synthesis, and we also assume this *P*2*Y*-receptor is activated by ATP. DAG in turn indirectly inhibits G-protein dependent PLC-*β* activation by catalyzing protein kinase C (PKC) activity. The activation of *IP*_3_ receptors by *IP*_3_ induces ERCa^2+^ release. We model this as a deterministic process, for simplicity, although the Ca^2+^ spiking that results resembles that which is observed experimentally (Skupin et al., 2008). As the cytosolic Ca^2+^ rises following *IP*_3_ receptor opening, DAG and Ca^2+^ promote PKC-dependent inhibition of PLC-*β* (Litosch, 2013). We also include the activity of ectonucleoside triphosphate diphosphohydrolase-1, also known as CD39. CD39 is expressed on the surface of microglial plasma-membrane and plays an important role in microglial migration by degrading ATP (Färber et al., 2008). We used the model of CD39-mediated hydrolysis of ATP into ADP and AMP from Kukulski *et al* to describe this degradation (Kukulski et al., 2005).

Many models for cell migration have been reported in the literature. These include models for actin polymerization (Mogilner and Edelstein-Keshet, 2002; Satulovsky et al., 2008), multi-cellular migration (De Pascalis and Etienne-Manneville, 2017), and tissue-level simulations of tumor growth (Van Liedekerke et al., 2019). For our model, we assumed *P*2*Y*12 triggers migration (Ohsawa et al., 2004) through the *P*2*Y*12/Akt axis (Irino et al., 2008). *P*2*Y*12 is activated by ADP (Haynes et al., 2006), upon which PI3K is agonized (Wu et al., 2007) through *G*_*i*/*o*_ signaling (Ohsawa et al., 2007). While, *P*2*Y*2 has also been shown to contribute to PI3K activation in the central nervous system (Weisman et al., 2012), we assumed for our model that *P*2*Y*12 is the predominant driver of PI3K, based on *P*2*Y*12^−/−^ data from (Ohsawa et al., 2007) that demonstrate an 80% reduction of migration relative to control. We interpolated data from Ohsawa *et al* to a distance of 4 µm following 5 minutes of stimulation. The *p*Akt-dependent migration rate was then fit to yield this short migration distance following integration of the *p*Akt levels over 5 minutes. We also introduced terms to reflect the Ca^2+^-dependent activation of CaM, which supports myosin activation *via* the MLCK pathway per Yao et al. (2013).

With respect to the ER Ca^2+^ content, we utilized ratiometric data from ATP-treated microglial cells presented by Ikeda et al. (2013) to estimate ER Ca^2+^ release *via* *IP*_3_ *R*. In that study, the authors obtained a series of Ca^2+^ transients to infer the distinct contributions made by ionotropic and metabotropic receptors. The authors utilized two ATP concentrations (100 μM and 1 mM) to activate P2X4 *versus* P2X7 receptors. For model validation, we converted the ratiometric data to concentrations by normalizing the cytosolic fluorescent intensity of resting microglia to 100 nM. We neglect *SOCE* given that this mechanism of Ca^2+^ entry occurs well after Ca^2+^ currents mediated directly by 5–10 min after ATP stimulation (Bianco et al., 2005; Ikeda et al., 2013; Adams et al., 2016; Jiang et al., 2017).

TNF*α* mRNA production following NFAT activation by Ca^2+^ was carried out according to Chun et al. (2019). Namely, we assume that Ca^2+^ mediated CaM/calcineurin activation promotes the translocation of NFAT into nucleus (Cooling et al., 2009), which in turn results in the transcription of TNF*α* mRNA (Hide et al., 2000).

#### 2.1.1 Numerical solution of the computational model

The resulting system of differential equations were numerically solved and optimized *via Python* (ver. 3.6) and Gotran (ver. 2020.2.0.), see (Chun et al., 2019) for details. As previously described (Stewart et al., 2018; Chun et al., 2019), the Generalized ODE (ordinary differential equation) Translator was utilized to implement the microglial model. The SciPy function, ODEINT, that employs the LSODA algorithm for stiff ODEs (Petzold, 1983) was used in the numerical integration of the microglia model. A time-step for the 10-min numerical integration was 0.1 m but the data is generally stored every second. These computations generate as output the time-dependent values of model ‘states’, such as intracellular Ca^2+^ or the open gates of the *P*2*X* channels. Model fitting was further tuned and refined by a genetic algorithm we developed (Stewart et al., 2018) to iteratively improve assigned parameters, such as the rate of Ca^2+^ leak and P2X4/P2X7 conductance. Parameters for the model components are summarized in Sect. S2. Based on these sets of parameters, our model outputs were Ca^2+^ transients with respect to ATP exposure duration and concentration, as well subsequent changes in other states including PI3K and Akt for which experimental reference data were available. Experimentally-measured outputs, such as Ca^2+^ transient decay time and amplitude, were used to optimize the model parameters by minimizing the error between model predicted outputs and experiment. In order to compare the Ca^2+^ transient data from Ikeda et al., we repeated the moving average and the variance.

All code written and simulation input files in support of this publication are publicly available at https://github.com/huskeypm/pkh-lab-analyses/tree/master/2021-P2Y-microglia. Generated data are available upon request.

### 2.2 Experimental details

#### 2.2.1 Microglia culture and imaging

For live cell Ca^2+^ imaging, 96 well plates were used for BV2 cell culture. Cells were plated in a density of 5,000 per well 2 days prior to imaging. On the day of Ca^2+^ imaging, the cells were incubated at 37°*C* with 5% *CO*_2_ with 1 *μg*/*ml* Fluo-4 AM (Thermo Fisher Scientific) in Leibovitz’s L-15 medium for 45 min. Fluo-4 was used to measure the intracellular Ca^2+^ concentration due to its lower binding affinity relative to indicators like fluo-8 (a.k.a. Fluo-2 medium affinity) (Hagen et al., 2012). This allows for measurements of large Ca^2+^ fluctuations with lower saturation and higher temporal resolution (Lock et al., 2015). Excess Fluo-4 AM was washed by Leibovitz’s L-15 Medium and again incubated in Leibovitz’s L-15 Medium for 30 min. A custom-built, wide-field, epifluorescence microscope having a ×10 objective with 488 nm laser was used for taking time lapse images. 20-min time lapse movies at 2 s intervals were taken for different concentrations of ATP (Thermo Fisher Scientific) within the first 10 min before adding ATP and the remainder after adding ATP.

For data in Figure 7, Ca^2+^ measurements were conducted at 0.5 Hz. For each ATP dose, approximately 48 cells were selected to calculate the Ca^2+^ transients (as indicated by pixel intensity). For each cell, the ATP-induced Ca^2+^ transient was normalized with respect to the control stage; namely, the average pixel intensity of the control stage was subtracted from the trace of the entire time course.

#### 2.2.3 Detection of ATP-induced Ca transients

The recorded images *via* the aforementioned Ca^2+^ imaging protocol were processed *via* custom python routines. The TIF file from the experiment was processed as a 3D matrix with a specific shape defined by (T,M,N) where *T* denotes the time-index, *M* × *N* was the image dimensionality (in the unit of pixels). The pixel intensity (gray-scale) was stored in 16-bit color depth. As the first step, the cell detection protocol identified cells from the image. Specifically, the intensity at each pixel of the image was summed up along the time-index to get a total image with shape (*M* × *N*). The total image was then subject to a log transformation and normalization. The histogram of pixel intensities of the normalized image was plotted to help identify a custom thresholding value. Parameters used for acquisition of these signals are embedded in the source code provided with this project. After determining the thresholding value, the normalized total image was converted into a binary image with intensities greater than the thresholding value identified as cell bodies with the rest as background. The detected cells in the binary image satisfying the aforementioned condition were segmented and labeled. Using this information, the original TIF gray-scale image (dark-field image) was utilized to record the change in the pixel intensity at each cell location, the average trace of which later represented the ATP-mediated Ca^2+^ transient of each cell.

We subsequently analyzed the Ca^2+^ transients before and after addition of different concentrations of ATP by taking time lapse images using widefield excitation. To normalize the data, we first calculated the average Ca^2+^ signal or fluorescence intensity in the control phase, the first 10 min without ATP. We then identified Ca^2+^ transients by dividing the entire time trace by the average fluorescence intensity. This was used to identify changes in the fluorescence signal.

##### 2.2.3.1 Detection of BV2 displacements

Using the bright-field data collected for ATP treated BV2 cells, we selected cells that exhibited displacements within the first 20 frames directly after ATP treatment, which corresponds to 600 s in total. To measure displacements, we manually selected reference points within a given BV2 cell process at the initial time point and its approximate position at the final (10th) frame, from which a vector was defined. The resolution of the brightfield data was approximately 1.6 µm/pixel, therefore the length in pixels of the displacement vector was converted into micrometers.

## 3 Results

### 3.1 Ca^2+^ waveform is simultaneously determined by P2 receptor and CD39 ectonucleotidase activity

**FIGURE 3:**
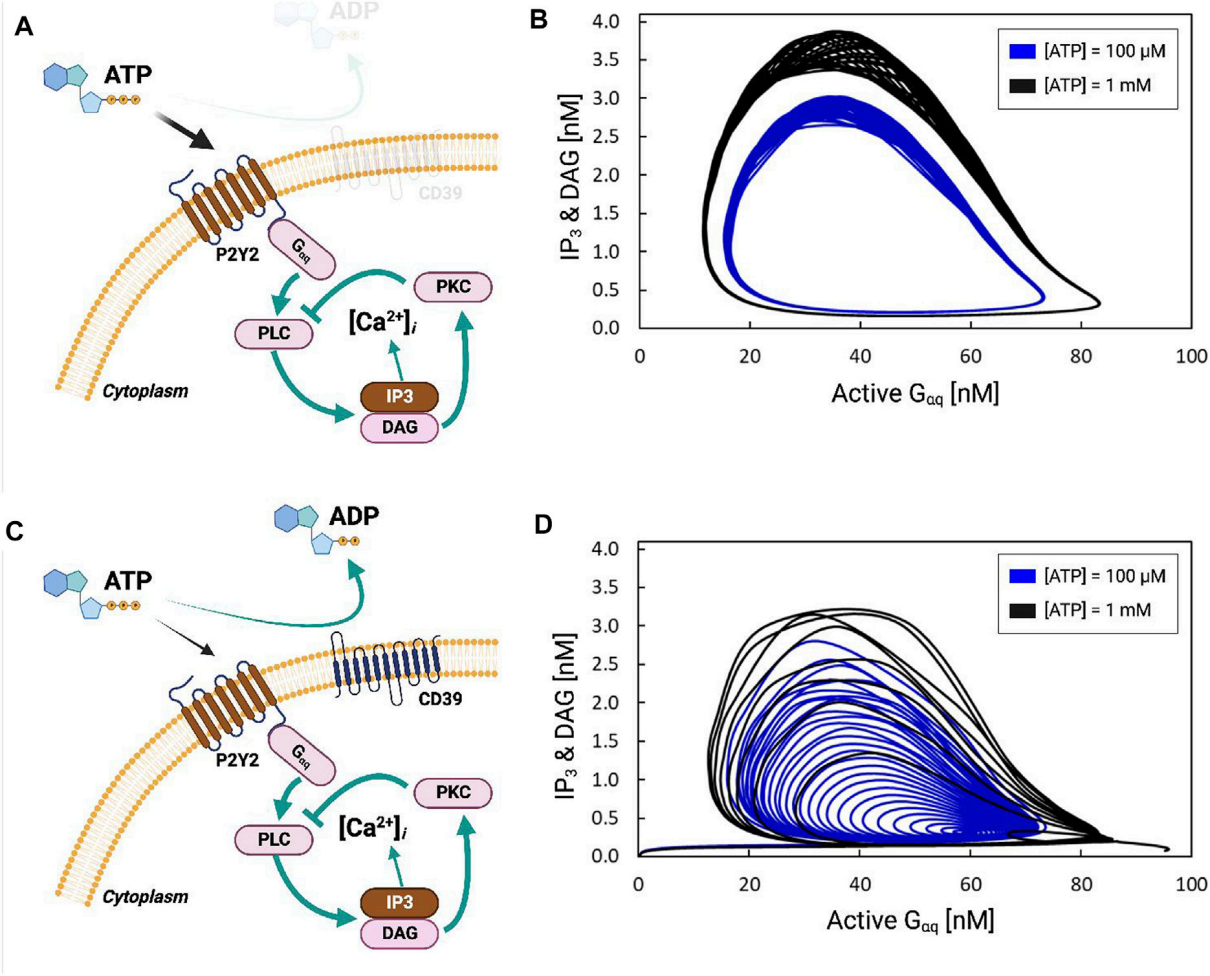
**(A)** Schematic of *P*2*Y*2-dependent activation of *IP*_3_ production *via* *G*_*αq*_. **(B)** *IP*_3_ and DAG concentration [nM] as a function of activated *G*_*αq*_ [nM] in response to 100 µM (black) and 1 mM (blue) ATP. The cyclic nature reflects that the concentrations are oscillatory. **(C,D)** are equivalent to **(A,B)**, except that the simulation includes the hydrolysis of ATP by the ectonucleotidase CD39.

**FIGURE 4:**
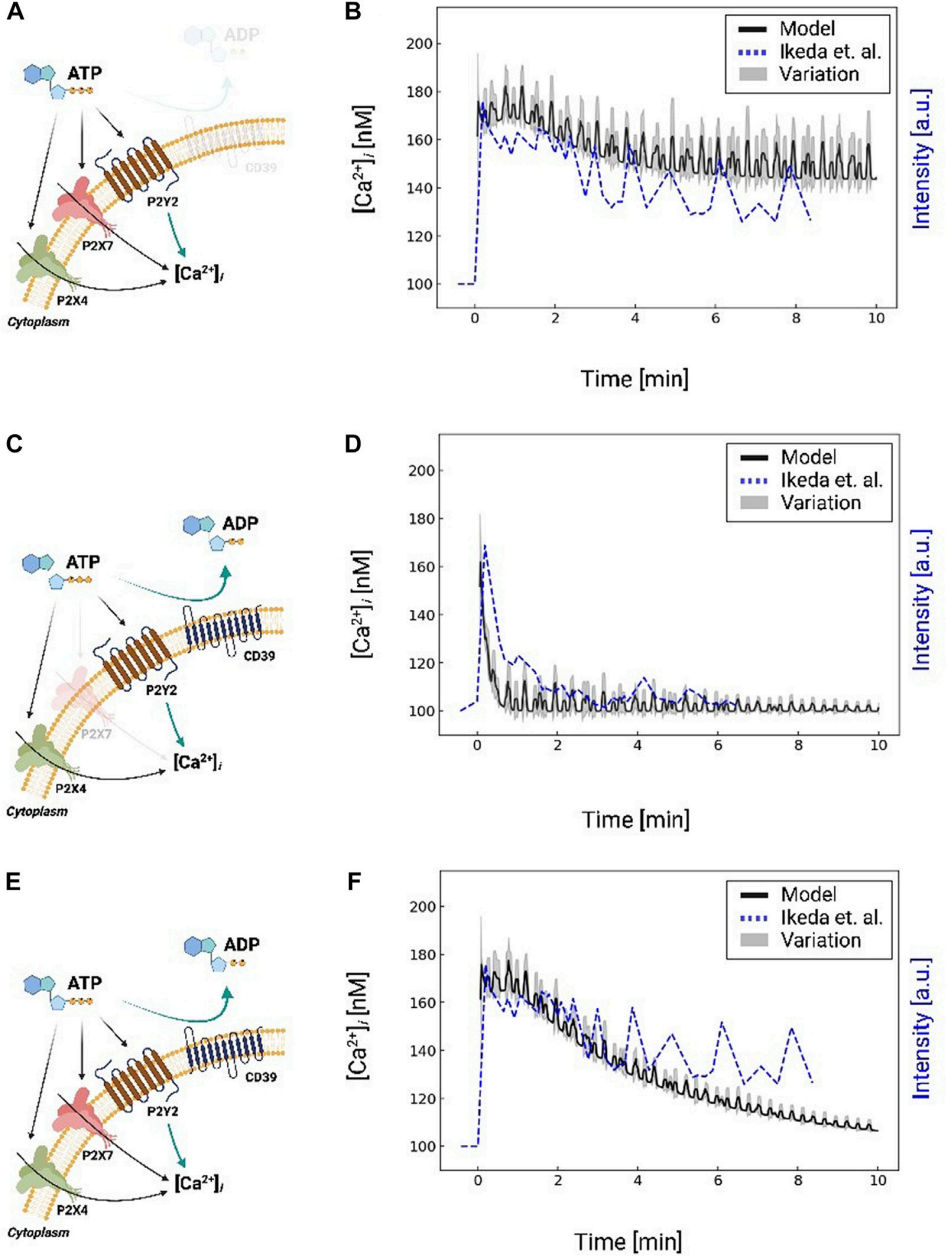
Schematic A for Ca^2+^ waveforms generated in a control system comprising P2X4, P2X7, and P2Y2 in response to 1 mM ATP for 10 min, but excluding CD39 nucleotidase activity. Schematic **(A)** is analogous to Schematic **(C)**, but includes CD39 nucleotidase degradation of ATP and excludes P2X7. Schematic C is analogous to Schematic E, but includes P2X7. Schematic **(E)** for Ca^2+^ waveforms generated in a control system comprising P2X4, *P*2*X*7, and *P*2*Y*2 and includes CD39 nucleotidase activity. Schematic **(B, D, and F)** comparison of predicted (black: moving average with a window size of 8) and are reported along the left *y*-axis. The experimentally-measured (Ikeda et al. (2013), dashed blue) Ca^2+^ transients are plotted against the right *y*-axis, corresponding to the schematic **(A, C, and E)**, respectively. Results excluding P2X7 and CD39 are shown in Supplementary Figure S2.

We developed a computer model of metabotropic (*P*2*Y*-class) and ionotropic (*P*2*X*-class) purinergic receptors to simulate Ca^2+^ signaling in microglia (Figure 2). These Ca^2+^ signals are induced by extracellular ATP and its metabolites, such as ADP, binding to P2 receptors (Kettenmann et al., 2011; Noda et al., 2012). We therefore expanded a published model of *P*2*X* receptor activation (Chun et al., 2019) to include *P*2*Y* contributions in order to investigate how both purinoreceptor classes influence intracellular Ca^2+^ transients. We first implemented and validated a model for G-protein mediated *IP*_3_ generation and *IP*_3_ receptor-mediated ER Ca^2+^ release contributed by Cuthbertson and Chay (1991) that we adapted to reflect *P*2*Y*-mediated Ca^2+^ transients (Figure 3). We then validated predicted Ca^2+^ waveforms generated by both *P*2*X*- and *P*2*Y*-class receptors against data collected in primary microglia by Ikeda et al. (2013), which provided the Ca^2+^ transients in microglia mediated by low and high ATP concentrations (100 µM and 1 mM, respectively. See Supplementary Figure S1; Figure 4). To align our simulation predictions with experiment, we additionally implemented a quantitative model of ATP degradation by the CD39 ectonucleotidase.

*P*2*Y*-driven *IP*_3_ signaling begins with the activation of *G*_*αq*_-protein, which stimulates Phospholipase C (PLC) to produce Inositol trisphosphate (*IP*_3_) and Diacylglycerol (DAG) from *PIP*_2_ (Figure 3A). This is followed by ER Ca^2+^ release *via* *IP*_3_-stimulated *IP*_3_ *R*s. Negative-feedback arises in this system as DAG produced by PLC-*β* activates PKC, which inhibits PLC-*β*. In Figure 3B, we demonstrate that the activation of *P*2*Y* by ATP and subsequently *G*_*αq*_ results in periodic fluctuations in DAG and *IP*_3_ concentrations. ATP concentrations of 100 μM and 1 mM were chosen for the simulations to be consistent with Ca^2+^ transient data reported in Ikeda et al. (2013). This is evident as stationary cycles in Figures 3A, B, where increases in active *G*_*αq*_ were accompanied by increases in *IP*_3_ and DAG; these increases continued until active *G*_*αq*_ was nearly saturated at 80 [a.u], whereafter *IP*_3_ and DAG rapidly decayed to zero as active *G*_*αq*_ was depleted. The stable oscillation shown in Figure 3B therefore resulted in periodic fluctuations of the Ca^2+^ concentration shown in Supplementary Figure S2B. Larger oscillations in *G*_*αq*_ activation and *IP*_3_ production were evident with 1 mM ATP relative to 100 µM. In contrast, including ATP hydrolysis *via* CD39 resulted in attenuated *G*_*αq*_ and *IP*_3_/DAG oscillations evidenced by cycles of decreasing size (see Figures 3C, D).

Stable *IP*_3_ oscillations are reported in many cell types and are a prototypical example of negative-feedback circuits (Tyson, 2002; Kekenes-Huskey et al., 2015). Interestingly, microglia exhibit both oscillatory and aperiodic Ca^2+^ transients, which suggests that the underlying *IP*_3_ synthesis is not exclusively periodic. Oscillations in feedback biochemical circuits are determined by the kinetics of the underlying enzymes, therefore non-oscillatory *IP*_3_ signals are theoretically possible and would manifest as single-peak Ca^2+^ waveforms (Ikeda et al., 2013). We demonstrate in Supplementary Figure S3 how variations in the parameters underlying *P*2*Y*-frequency dependent *G*_*αq*_ activation can yield stable oscillations *versus* aperiodic behavior. Namely, by reducing the input parameter *k*_*g*,*cc*_ that controls the rate of *G*_*αq*_ activation, the system reverts to non-oscillatory behavior. Similar effects can be shown by varying other parameters describing the *G*_*αq*_ negative-feedback circuit, which suggests that the activity of proteins comprising the *G*_*αq*_/*IP*_3_ signaling pathway determine whether *IP*_3_ and thereby Ca^2+^ are oscillatory *versus* non-oscillatory.

*IP*_3_ invokes intracellular Ca^2+^ release from the endoplasmic reticulum *via* *IP*_3_ receptors. We therefore fit the model’s predicted *IP*_3_-induced Ca^2+^ transients to reproduce experimental data collected by Ikeda et al. (2013). Namely, we fit the initial peak amplitude ([Ca^2+^]_*i*_ = 180 nM) to match the Ikeda *et al* data in MG5 microglial cells treated with 1 mM ATP (Ikeda et al., 2013), for which P2X7 was knocked-out to isolate the contributions of *P*2*Y*12 and P2X4 (see Supplementary Figure S2). We reflected this condition in our overall Ca^2+^ signaling model by zeroing the P2X7 currents. In Figure 4B, we compare our predictions of *IP*_3_-mediated Ca^2+^ release following a 10-min 1 mM ATP treatment (black) relative to the experimentally-measured transients from Ikeda *et al* (dashed blue). After this initial peak, our model predicts an oscillatory Ca^2+^ waveform that is complemented by decreases in ER Ca^2+^ owing to *IP*_3_ receptor activation (Supplementary Figure S4). The predicted Ca^2+^ waveform resembles the oscillations observed by Ikeda *et al*; however, our oscillations do not decay with time.

While a variety of mechanisms could be attributed to this discrepancy, such as the desensitization of *P*2*Y* receptors to ATP (Jacobson et al., 2006), we speculated that the availability of ATP for triggering *P*2*Y* was the prominent source of error. Extracellular ATP is rapidly degraded into ADP and AMP by ectonucleotidases (Kukulski et al., 2005), of which CD39 is the primary isoform in microglia that degrades ATP (Färber et al., 2008). To represent this contribution, we implemented a mathematical model from (Kukulski et al., 2005) to simulate CD39-catalyzed hydrolysis of ATP into ADP and AMP. Our implementation is validated against experimental data in Supplementary Figure S5, for which nucleotide concentrations were measured in COS-7 cells over a 1-h time interval (Kukulski et al., 2005). Importantly, these data demonstrate that the ATP pool was depleted within minutes; this depletion was accompanied by a transient ADP pool that was maximal at t = 4 min and subsequently decayed to zero. After including ectonucleotidase contributions in our microglia model, the predicted cytosolic Ca^2+^ transients decayed in a manner consistent with experimentally measured data (Figure 4D) without additional fitting. Hence, our simulations indicate that the ectonucleotidase CD39 plays a prominent role in shaping the Ca^2+^ waveform by controlling the nucleotide pool available to purinoreceptors.

After validating our model of *P*2*Y*-induced Ca^2+^ dynamics, we restored P2X7 receptor contributions and compared model predictions against analogous experiments in Figure 4F. We again simulated the system subjected to 1 mM ATP for 10 minutes with and without ectonucleotidase activity (shown in Figures 4C,D; Supplementary Figure S2 respectively). In contrast to the P2X7 −/− data, we observed a modestly higher peak Ca^2+^ transient amplitude that was followed by a prolonged plateau as would be expected from P2X7 currents (Egan and Khakh, 2004). Predicted Ca^2+^ oscillations decayed toward resting Ca^2+^ levels after approximately 10 minutes, albeit at a faster rate than observed experimentally. We report similar findings upon 100 µM ATP treatment in Fig. S1, which is sufficient to activate P2X4 (Jacobson et al., 2006), but not P2X7. Both the predicted and experimentally measured transients have an initial spike that rapidly decays. The data from Ikeda *et al* show a slower spike in Ca^2+^ than our prediction. Altogether, our data suggest that a diverse ensemble of Ca^2+^ waveforms are invoked by controlling P2 receptor activation and nucleotide availability.

### 3.2 P2 mediated Ca^2+^ waveforms contribute to migration

**FIGURE 5:**
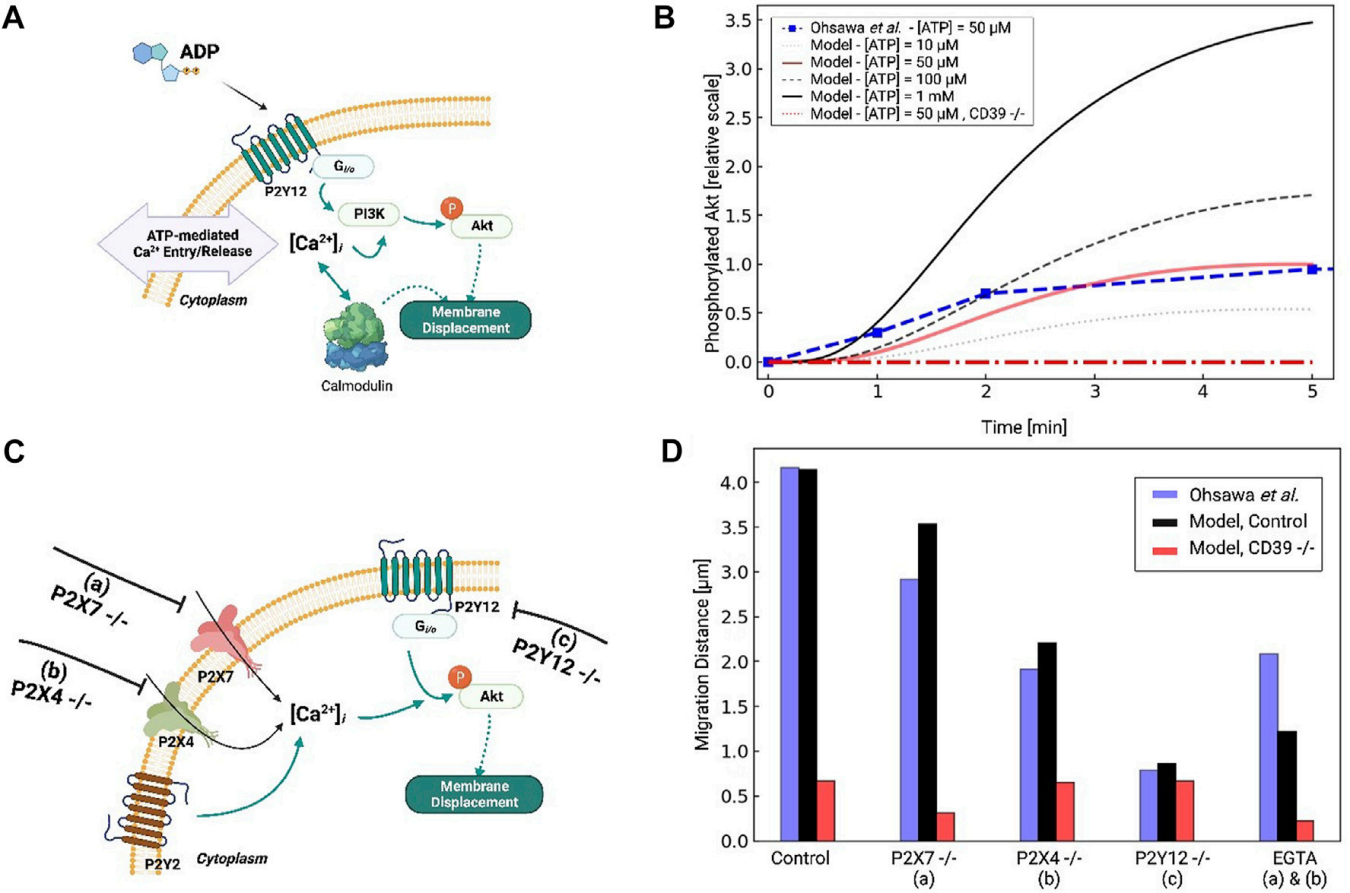
**(A)** Schematic of Akt phosphorylation *via* *P*2*Y*12- and *P*2*Y*-mediated Ca^2+^ signaling pathways in response to ATP. **(B)** Predicted *p*Akt level as a function of time in response to 50µM to 1 mM ATP applied for 5 min. Experimental data for 50 µM ATP from Ohsawa et al. (2004) are presented in red. **(C)** Schematic of *P*2*Y*12- and Ca-mediated migration in response to ATP, assuming control, *P*2*X*7 −/− **(A)**, P2X4 −/− **(B)** and *P*2*Y*12 −/− **(C)** conditions. **(D)** Predicted migration distances (black) *versus* experimental measurements by Ohsawa *et al* under control and a-c conditions.

We next examined how *P*2*Y* activation and *P*2*Y*-mediated Ca^2+^ waveforms control cell migration and displacement (see Figure 5). *P*2*Y*12 activation is essential for chemotactic migration and rapid membrane displacements in ATP-stimulated microglia (Ohsawa et al., 2004; Ohsawa et al., 2007; Irino et al., 2008; Kettenmann et al., 2011). *P*2*Y*12 receptors primarily activate the G-protein family *G*_*i*/*o*_. and thereby promote Akt phosphorylation indirectly *via* PI3K to yield *p*Akt, which in turn activates pathways underlying cell migration in microglia.

We first fitted our *p*Akt responses under 50 µM ATP treatment to reproduce data from Ohsawa *et al* in neonatal Wistar rat microglia. Since their data were obtained by Western blot, we normalized *p*Akt levels relative to the maximum recorded in their experiments for our model. The resulting fit is shown in Figure 5B, which indicates maximal *p*Akt levels were obtained at about 4 min and were in close agreement with the experiment. *P*2*Y*12 activation was maximal at this time given that its primary agonist, ADP, is maximal at 4 min due to ectonucleotidase activity (see Supplementary Figure S5). We also predict that Akt phosphorylation increased with increasing ATP concentration, as 100 μM and 1 mM ATP treatments resulted in 50% and 300% increases in *p*Akt relative to 50 µM.

The phosphorylation of Akt has been shown to depend on extracellular Ca^2+^. This is supported by evidence suggesting that the inhibition of *P*2*X* receptors (PPADS and TNP-ATP for P2X7 and P2X4, respectively) and removal of extracellular Ca^2+^ *via* excess EGTA all reduce *p*Akt levels (Ohsawa et al., 2004; Ohsawa et al., 2007; Irino et al., 2008). We therefore modified our computational model to include Ca^2+^-dependent PI3K/*p*Akt activation, as a step toward investigating the extent to which purinoreceptor-encoded Ca^2+^ waveforms influence Akt activation and subsequent migration (Divolis et al., 2016). To determine the Ca^2+^ dependence of PI3K activation, we referred to data from Ohsawa et al. (2004) that reported Akt phosphorylation following *P*2*Y*12, P2X7, or P2X4 inhibition. Under *P*2*Y*12 inhibition, they observed a 90% reduction in *p*Akt upon treatment of 50 µM ATP for 5 min relative to WT. We attributed the remaining 10% of the *p*Akt phosphorylation to Ca^2+^ influx from the *P*2*X* receptors. This was motivated by our observations that 1) P2X4 in particular generated prominent Ca^2+^ transients with micromolar ATP treatments (Ohsawa et al., 2007) and 2) that EGTA treatment nullified *P*2*X*-mediated Ca^2+^ transients and significantly reduced PI3K activation (Ohsawa et al., 2007). Indeed, we predict in Supplementary Figure S6B that EGTA treatment reduced *p*Akt by 75% normally conducted by *P*2*X* receptors.

We next examined the effects of P2X4 and P2X7 inhibition on PI3K activation, and subsequently, *p*Akt. Without additional refitting, our model predicted 40% and 15% reductions in *p*Akt levels solely from P2X4 and P2X7 inhibition, respectively. Our result for P2X4 was in close agreement with Ohsawa (see Fig. S6A and B). These substantial reductions upon nullifying P2X4 contributions therefore implicate this ionotropic receptor in phosphorylating Akt. We additionally verified that *P*2*Y*12 −/− all but eliminates Akt phosphorylation. Lastly, we predict that blunted ectonucleotidase hydrolase function enhances *p*Akt phosphorylation, which suggests that prolonged Ca^2+^ waveforms further promote Akt activation (Ohsawa et al., 2004) (shown in Figure 5B).

*P*2*Y*12, PI3K, Akt, and extracellular Ca^2+^ control microglia migration behavior (Ohsawa et al., 2004; Ohsawa et al., 2007). This is in part supported by data from Ohsawa *et al* demonstrating microglia with inhibited PI3K exhibit negligible migration when treated with ATP (Ohsawa et al., 2004). We therefore assumed microglia migration rates were proportional to *p*Akt levels in accordance with data from Ohsawa et al. (2004). Those data suggested that ATP-treated microglia migrate distances of approximately 48 microns following 1 hour of 50 µM treatment. We tested our fitted model by reporting migration distances upon inhibition of P2X4, P2X7, and *P*2*Y*12 (see Figure 5C). Without additional fitting, our simulated data nearly reproduced the 75% reduction in migration following *P*2*Y*12 −/− that was reported by Ohsawa *et al* owing to reduced *p*Akt. An intermediate reduction in migration distance following P2X4 inhibition was also comparable to data from Ohsawa *et al* (Figure 5D). Our model additionally predicts that reducing ectonucleotidase activity decreases migration, which is consistent with experimental data for CD39 −/− microglia (Färber et al., 2008). This is because ATP cannot be degraded into ADP, which is necessary for P2Y12 activation. Importantly, these simulated data confirm that *P*2*Y*12 inhibition, which was expected to all but eliminate Akt phosphorylation, dramatically reduced, but did not entirely eliminate migration. Together with the reductions in migration following *P*2*X* inhibition, these data implicate a role of extracellular Ca^2+^ in mediating Akt activation and migration.

### 3.3 Ca^2+^ waveforms and their impact on migration *versus* cytokine responses

**FIGURE 6:**
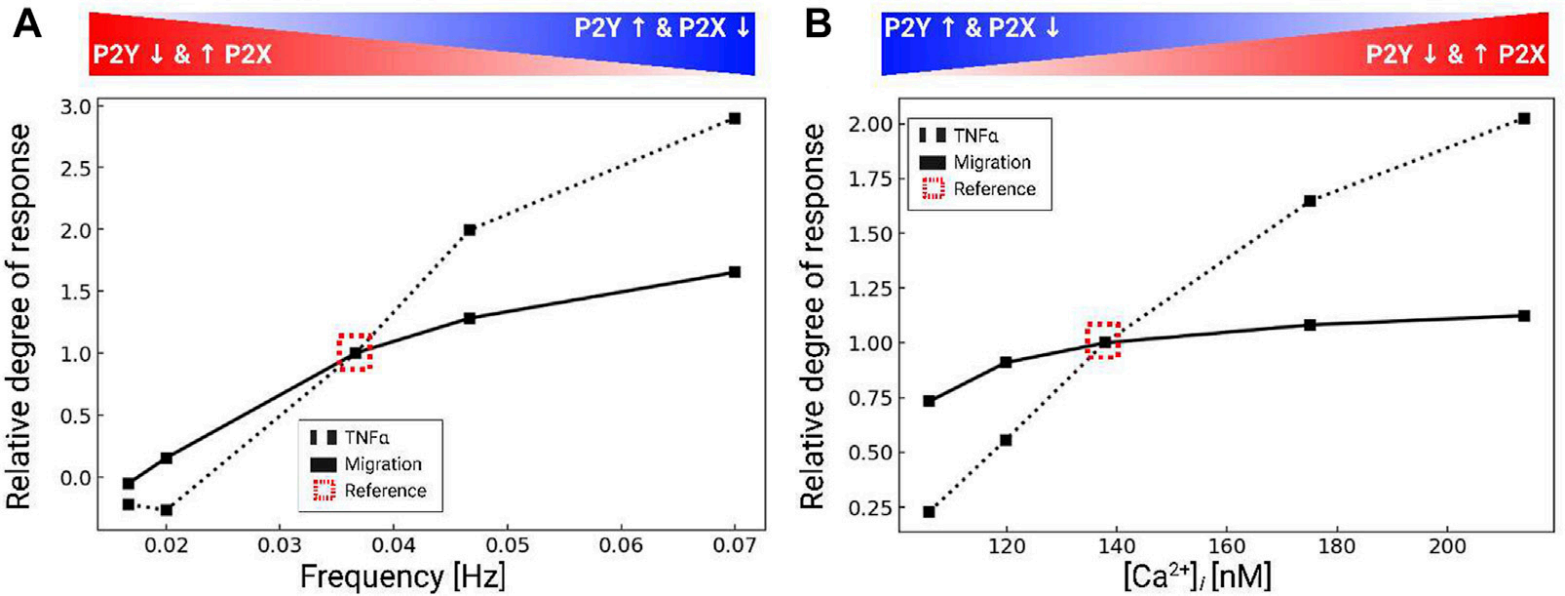
Predicted TNF*α* production (black dashed) and migration (black solid), normalized to control conditions (dashed box) in response to variations of the intracellular Ca^2+^ waveform frequency **(A)** and amplitude **(B)**. All calculations were performed using 200 µM ATP and the data were collected over 5 min after ATP addition.

Our data thus far indicate that microglia have robust Ca^2+^ responses to P2 receptor activation that influence migration. We previously showed in (Chun et al., 2019) that cytokine synthesis and release in microglia were driven by intracellular Ca^2+^ signaling. This raised the question as to how ATP-induced Ca^2+^ waveforms determined migration *versus* inflammatory cytokine responses in microglia. To answer this question, we also predicted migration distances as a function of Ca^2+^ waveform amplitude and oscillation frequency when subject to 200 µM ATP for 5 min in Figure 6. 200 µM ATP was chosen to afford better control in modulating the frequency and amplitude of the Ca^2+^ waveforms. The amplitudes and frequencies were controlled by changing the *IP*_3_ pathway parameter *k*_*g*,*cc*_. As a measure of cytokine responses, we report predicted TNF*α* released levels, using our validated model from (Chun et al., 2019). For reference, the dashed box indicates baseline migration and TNF*α* responses for the model when subject to 200 µM ATP. Per (Hide et al., 2000), secreted TNF*α* was undetectable under 1 h and reached a maximum concentration (425 pg/10^6^ cells) for 3 mM ATP after 6 h. Our model predictions indicate that both migration and TNF*α* increase with increasing Ca^2+^ waveform frequency and amplitude (Figure 6). Although TNF*α* responses were predicted to increase at a greater rate than migration for increasing Ca^2+^ waveform frequency and amplitude, we anticipate produced TNF*α* would nonetheless be undetectable at 5 min. This is based on observations by Hide *et al* that minimal TNF*α* (10 pg/10^6^ cells) was measured at 1 h, even with ATP treatments exceeding 1 mM (3 mM). Importantly, our model demonstrates that 1) both migration and TNF*α* production were positively correlated with the frequency and amplitude of the Ca^2+^ waveform and 2) migration could be triggered without significant TNF*α* responses when the Ca^2+^ waveforms were of short duration, low amplitude and low frequency.

### 3.4 ATP-dependent responses in different microglia phenotypes

**FIGURE 7:**
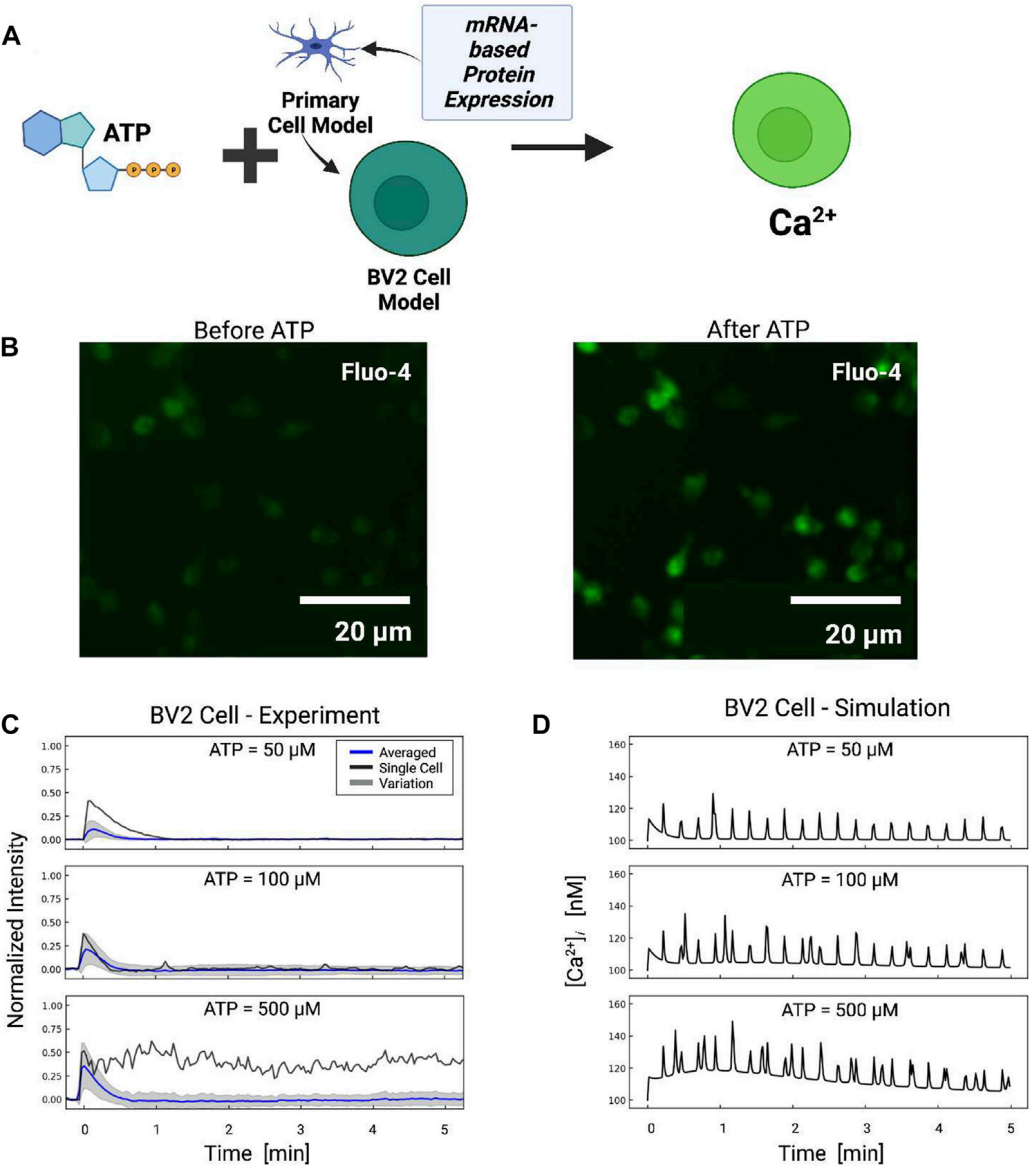
**(A)** Schematic of ATP-induced Ca^2+^ transients in BV2 cell simulations assuming P2 expression levels inferred from BV2 cell mRNA (He et al., 2018). **(B)** Ca^2+^ transients measured in BV2 microglia before and after ATP treatment. **(C)** Experimentally measured (blue) and predicted (red) Ca^2+^ transients from BV2 microglia. The shaded area represents the variation in signals measured in all cells. Oscillatory calcium signals were observed and a representative example of which is reported for each ATP dose (black). Transients recorded in each cell are reported in Fig. S7. **(D)** Predicted transients in primary microglia for comparison.

Microglia and immortalized microglia cell lines assume diverse phenotypes that are accompanied by differences in purinoreceptor expression (Bianco et al., 2005; Crain and Watters, 2015; He et al., 2018). As a proof-of-principle, we tested relative to the primary microglia used as a basis for our model how changes in purinoreceptor expression exhibited in the BV2 microglia cell line could influence their ATP-triggered Ca^2+^ waveforms and Ca^2+^-dependent functions. This cell line was chosen, given that these cells are easier to culture and characterize compared to primary cells. Although mRNA expression levels do not necessarily directly correlate with protein expression (Erb and Notredame, 2016), as a first approximation we proportionately rescaled the purinergic receptor responses in our model according to the relative change in mRNA expression (see Table 1). We adjusted P2 contributions in our model in accordance with mRNA data sets published for P2X4, *P*2*X*7, *P*2*Y*2 and *P*2*Y*12 (see Figure 7) in primary relative to BV2 microglial cells (He et al., 2018). Those data reflect 5-fold and 2-fold reductions in P2X4 and P2X7 mRNA relative to primary cells, no change in *P*2*Y*2, and a near complete elimination of *P*2*Y*12 mRNA.

**TABLE 1:** Relative expression of P2 receptors in acutely isolated, primary cultured microglia, and BV2 cells in relative scale. Reported receptor mRNA expression is normalized to the expression level (mRNA count) found in primary cultured microglia and are incorporated in our model as scaling factors for receptor concentration (*ρ*_*P2X4*_, *ρ*_*P2X7*_, *ρ*_*P2Y2*_, and *ρ*_*P2Y12*_). *The mRNA expression was acquired from the comparison between cultured mouse microglia and BV2 cells (He et al., 2018).

Receptors	mRNA expression	
	Primary	BV2*
*P*2X4	1×	0.18×
*P*2X7	1×	0.65×
*P*2*Y*2	1×	1.02×
*P*2*Y*12	1×	0.030×

#### 3.4.1 BV2 Ca^2+^ transients

Based on the adjusted P2 responses, and without any additional fitting, we predicted Ca^2+^ transients in response to 50–500 µM ATP treatment applied for 10 min (Figure 7). The model demonstrated that the reduced *P*2*X* expression implied for BV2 cells resulted in moderately smaller Ca^2+^ transients relative to primary cells. The largest reductions were predicted at 50 and 100 μM, which was consistent with the preferential activation of P2X4 with micromolar ATP and the channel’s five-fold reduction in mRNA *versus* primary microglia. In contrast, modest reductions of 15% in Ca^2+^ transients after 20 s were predicted at 500 μM, which was inline with the P2X7 channel’s 35% reduction in P2X7 mRNA expression. Oscillations stemming from *P*2*Y*2 activation were predicted in the BV2 line and commensurate with those from primary cells.

To validate these model predictions, we measured Ca^2+^ transients in cultured BV2 cells. Because we did not have calibrated BV2 Ca^2+^ data, we assumed the peak Ca^2+^ amplitudes at 50 µM were approximately 112 nM in amplitude to be consistent with the 82% reduction P2X4 mRNA. We report in Figure 7 the average Ca^2+^ transients (blue) for the BV cells, while the cell-to-cell variance is represented by gray shaded regions. We found that the initial phase ( < 1 min) of the predicted Ca^2+^ transients at 50, 100, and 500 µM exhibited a rapid spike comparable to the experimental data. The averaged fluorescence data matched the fluctuations predicted by our model, however, the individual traces exhibited oscillations (Supplementary Figure S7). Overall, it was evident from our model predictions that the expression differences in *P*2*X* channel mRNA were sufficient to reproduce the initial phase of the experimentally-measured Ca^2+^ transients and capture Ca^2+^ oscillations evident in a subset of BV2 cells. However, additional measurements of protein mRNA or expression levels of *P*2*Y*2 or downstream targets would ultimately be necessary to align the model predictions with experimental observations.

##### 3.4.1.1 BV2 migration

**FIGURE 8:**
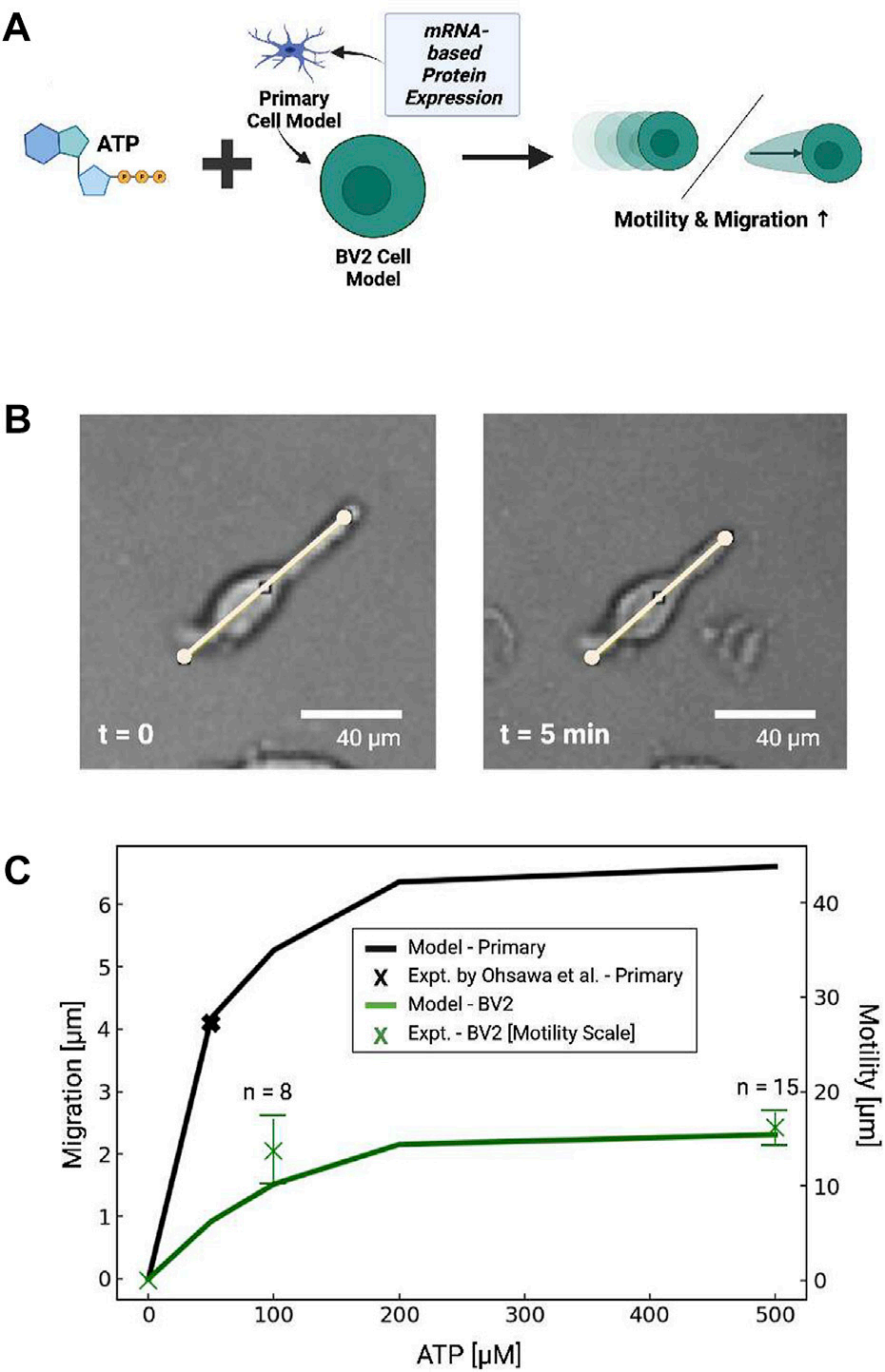
**(A)** Schematic of predicted migration or displacement in primary microglial cells or cells with P2 expression estimated from BV2 cell mRNA (He et al., 2018). **(B)** Bright field image demonstrating ATP-dependent displacement of microglial cell membrane (100 µM ATP at 5 min) **(C)** Predicted (lines) migration or displacement distance following 5 min ATP treatment (0–500 µM) in primary microglia (blue) and BV2 cells (green). Experimental data in primary cells from Ohsawa et al. (2004) and measured in BV2 cells are shown by black and green X’s, respectively, where the bars represent standard error.

We last predicted BV2 migration upon 5 min ATP treatment, based on the assumptions of reduced *P*2*X* and *P*2*Y*12 expression (see Figure 8). In accordance with the reduced *P*2*Y*12 mRNA measured in BV2 cells, across all ATP concentrations we predicted a nearly 70% reduction in migration relative to primary cells. This reduction was consistent with the *P*2*Y*12 −/− data reported by Ohsawa *et al* that demonstrated reduced, but not entirely eliminated, migration in primary microglia. The predicted distances monotonically increased with higher concentrations of applied ATP, which was consistent with the Ca^2+^ dependency in migration exemplified in Figure 6.

To validate these predictions, we examined subsets of BV2 cells that exhibited linear extensions of their membrane akin to the cellular processes evident in branched microglia (Figure 8B). We found that these cells rapidly contracted upon ATP treatment, as exemplified in Figure 8, but otherwise we did not observe appreciable directed migration over the data collection interval. We therefore defined this movement as the displacement of plasma membrane following ATP treatment. To assess ATP-dose dependencies for these responses, we measured the displacement of these extensions along manually-defined vectors. We report in Figure 8C that maximum displacement distance of 1.6 × 10^1^µm was evident in response to 500 µm ATP, indicating the displacements increased with increasing ATP. Further, the dose-dependent displacement rates were consistent with the migration distances we predicted for BV2 cells in Figure 8. Although these displacements were only reflected in a minority of the imaged cells, we found that morphological changes, surface ruffling and minor displacements of the cell somas were evident in a larger number of cells by visual inspection. These effects were more prominent for ATP-treated cells than those not treated with ATP (ATP = 0 µM).

## 4 Discussion

### 4.1 Findings of this paper

**FIGURE 9:**
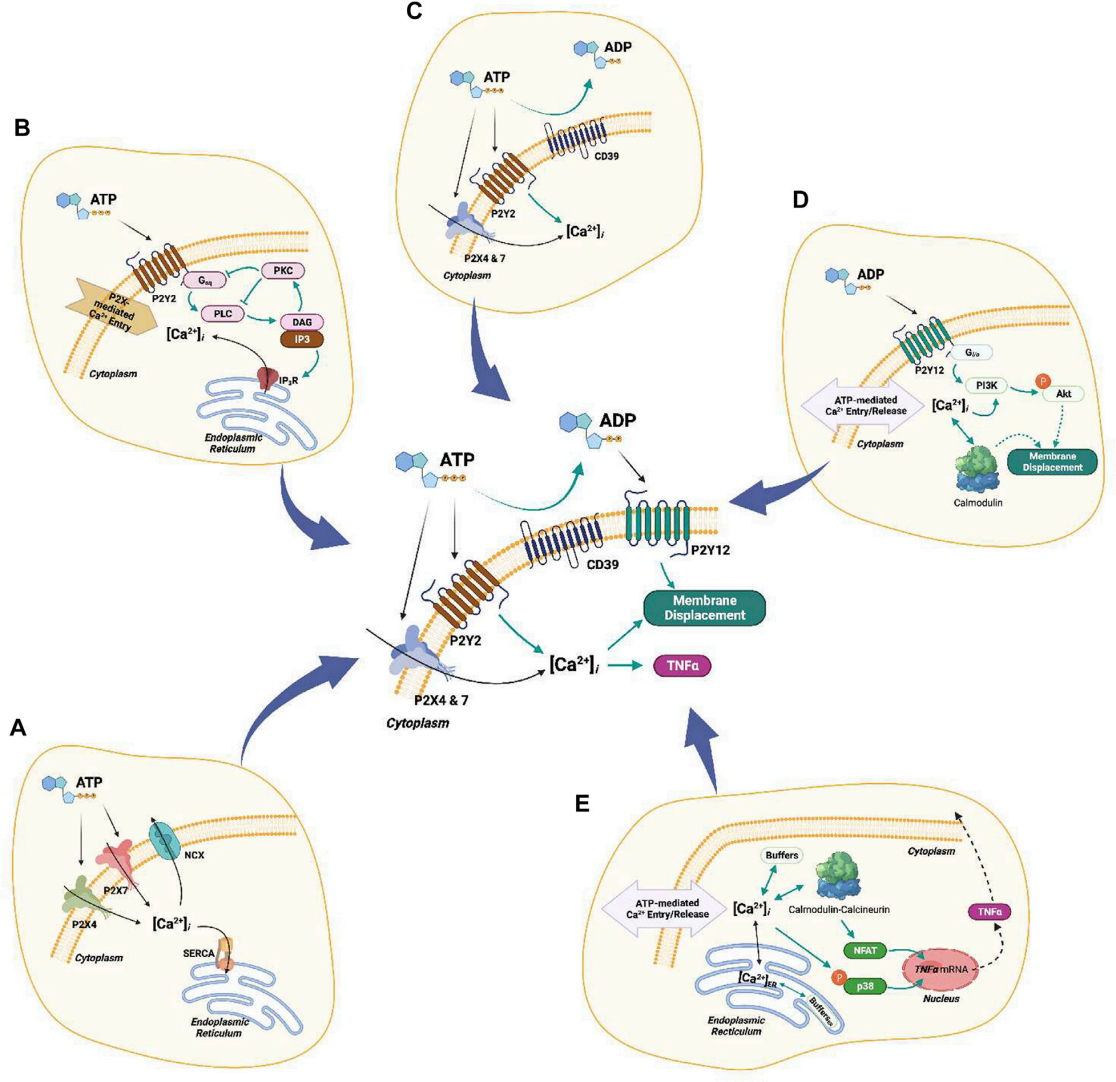
Complete schematic of the computational model. **(A)** *P*2*X*4/7-mediated Ca^2+^ entry. **(B)** *P*2*Y*2-mediated Ca^2+^ transients. **(C)** Hydrolysis of ATP by ENTs (CD39). **(D)** *P*2*Y*12-mediated cell migration. **(E)** Ca^2+^-dependent downstream cascades associated with TNF*α* production.

In this study we used computational modeling to investigate how *P*2*X* and *P*2*Y* receptors collectively regulate Ca^2+^ signaling and migration in microglia in response to ATP. This investigation extended a computational model of ionotropic *P*2*X* receptor activation (Chun et al., 2019) to incorporate contributions from metabotropic *P*2*Y* receptors. A schematic of the resulting model is shown in Figure 9. With this model, we:

1. Determined the extent to which P2 receptor activation in microglia shaped the waveforms of intracellular Ca^2+^ signals
2. Assessed the effects of *P*2*Y*12 activation on the PI3K/Akt axis that drives microglia migration.
3. nvestigated the extent to which mRNA data from BV2 microglia could be used to predict ATP-dependent responses in other microglia cell types.

### 4.2 Purinergic receptors and ectonucleotidases collectively control microglial Ca^2+^ waveforms

**FIGURE 10:**
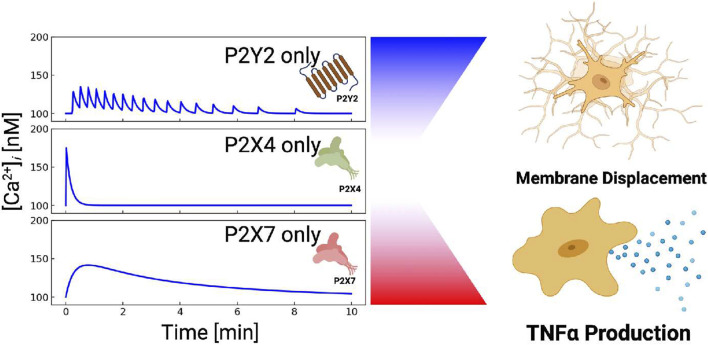
P2X and P2Y receptors can be combined to control different cellular outcomes. P2X4-induced Ca^2+^ entry manifests a sharp but brief rise in cytosolic Ca^2+^. P2X7-mediated Ca^2+^ entry results in blunt and prolonged Ca^2+^ elevations. *P*2*Y*-class receptors (mainly *P*2*Y*2) yield oscillatory Ca^2+^ transients, while *P*2*Y*12 (not shown) promotes membrane displacement.

Our first goal was to determine the extent to which highly-expressed P2 receptors in microglia shaped the waveform of intracellular Ca^2+^ signals in response to ATP. Indeed, our model indicated that metabotropic *P*2*Y* receptors contribute significantly to ATP-induced Ca^2+^ signals in microglia. Since *in vivo* microglia typically express high levels of *P*2*Y* receptors (Crain et al., 2009), this further suggests that ER Ca^2+^ release may play a more significant role in tissue microglia than would be observed in *ex vivo* cultured microglia that are more commonly studied. Further, unlike the *P*2*X* receptors that generally present high-amplitude, single-peak Ca^2+^ waveforms, we show that *P*2*Y* receptors can adopt oscillatory or transient, single-peak, Ca^2+^ fluctuations (shown in Fig. S3). Given that sustained increases in basal intracellular Ca^2+^ levels are associated with pathological states (Hoffmann et al., 2003; Tvrdik and Kalani, 2017) and spontaneous oscillations are typical of homeostatic cells (Hoffmann et al., 2003; Kettenmann et al., 2011), the balance of *P*2*X* *versus* *P*2*Y* contributions may be indication of the cell phenotype (Figure 10). Additionally, the ability for *P*2*Y* to encode diverse oscillatory and nonoscillatory signals could serve as a mechanism for controlling Ca^2+^-dependent functions in microglia. Our speculation is consistent with findings in other Eukaryotic cells that the dynamic profiles of Ca^2+^ waveforms tune cellular outcomes (Smedler and Uhlén, 1840). As examples, oscillatory Ca^2+^ waves in oocytes are observed during fertilization, while the timing of Ca^2+^ pulses in cardiac myocytes can selectively activate rapid CaMKII- *versus* slow NFAT-mediated gene responses (Zhang et al., 2007).

Although we did not investigate subcellular Ca^2+^ dynamics in microglia, the spatial distribution of Ca^2+^ transients is likely as important as their temporal character. To our knowledge, investigations of subcellular Ca^2+^ dynamics in microglia are fewer in number compared to other cell types, including muscle cells (Hake et al., 2012). Nonetheless, a recent example with ATP-treated microglial revealed spatially heterogeneous Ca^2+^ profiles (Venkateswarlu et al., 2020). This heterogeneity could result either directly from non-uniform distributions of ionotropic receptors conducting Ca^2+^, or from second messengers like *IP*_3_ that trigger localized ER Ca^2+^ release. Future experiments that characterize localized Ca^2+^ transients in microglia will be necessary to assess their functional significance relative to more uniform global signals.

Our simulations indicate that the activity of proteins belonging to the *G*_*αq*_/*IP*_3_ pathway determine whether *P*2*Y* receptors generate single, long duration peaks or oscillations. *P*2*Y* receptors are GPCRs, of which *P*2*Y*1, *P*2*Y*2, *P*2*Y*4, *P*2*Y*6, and *P*2*Y*11 promote *IP*_3_-dependent ER Ca^2+^ release *via* activating *G*_*αq*_ proteins (Kettenmann et al., 2011). This pathway includes PKC-dependent negative-feedback inhibition of PLC*β* (Litosch, 2013), which gives rise to stable *IP*_3_ oscillations and periodic intracellular Ca^2+^ release (Skupin et al., 2008). Negative feedback inhibition is a property of classical biochemical oscillators, for which the enzyme reaction rates determine the periodicity and decay of products like *IP*_3_ (Tyson, 2002), Although these oscillations may be stochastic, deterministic representations (Sneyd et al., 1995; Tyson, 2002) amenable to ordinary differential equation modeling are commonly used, given their ability to approximate the amplitude and peak-to-peak intervals of experimentally-measured Ca^2+^ release events (Cao et al., 2014). Since these parameters represent the activity of proteins composing the *IP*_3_ synthesis pathways, waveforms exhibited in microglia are expected to be inherently sensitive to factors such as protein expression and co-localization (Kekenes-Huskey et al., 2015).

Our simulations strongly implicate the role of ectonucleotidase activity in controlling the responsiveness of microglia to extracellular ATP and related nucleotides. We demonstrate that neglecting ectonucleotidase activity in our model yielded sustained Ca^2+^ oscillations that were inconsistent with measurements in microglia and specifically the data collected by Ikeda et al. (2013) for MG5 microglial cells. Although we assume that CD39-dependent degradation of ATP contributes to the decay of the *P*2*X*-driven Ca^2+^ transients with time, a similar response may also result from *P*2*X* receptor desensitization (Koshimizu et al., 2000) or changes in trafficking (Robinson and Murrell-Lagnado, 2013). We believe our assumption of CD39 degradation is reasonable, given that a related hematopoietic cell type exhibited pronounced Ca^2+^ transients mediated by P2X7 only when CD39 was inhibited by the nonhydrolyzable *βγMeATP*. In other words, blockade of nucleotidase activity increased the ATP available to stimulate the purinoreceptors. This therefore suggests that ectonucleotidase activity helps determine the duration of ATP-mediated Ca^2+^ waveforms in microglia and ultimately cellular responses like migration (*via* ADP) and cytokine production. The ectonucleotidases CD39 and CD73 are the most highly expressed nucleotidases in microglia (Matyash et al., 2017). CD39 rapidly hydrolyzes ATP and ADP into AMP, which curtails Ca^2+^ waveforms within minutes of ATP treatment in our simulations. While our model of CD39 activity was parameterized to fit data from Robson et al. (2006), more detailed models such as from Sandefur et al. (2017) could give additional insights into how other expressed ectonucleotidases influence microglial responses to ATP. As an example, augmenting the CD39 model with contributions from the CD73 ectonucleotidase isoform (Zimmermann, 1992), which metabolizes AMP into adenosine, will help determine which adenine metabolites predominate at the cell surface (Rahmaninejad et al., 2020), as well as the receptors they stimulate.

Interestingly, it is increasingly recognized that extensions of the microglia plasma membrane exhibit physical contacts with neural synapses (Schafer et al., 2012), which may be implicated in how microglia prune neural synapses (Stogsdill and Eroglu, 2017). ATP can be intermittently released in these junctions (Robson et al., 2006; Harris et al., 2012), therefore we expect that the timescale and amplitude of those release events, as well as the rate by which ATP is metabolized, will control how microglia respond to these intercellular signals to fulfill their homeostatic functions. Here, spatially-explicit models of nucleotidases (Rahmaninejad et al., 2020) that predict local ATP pools between interfaced cells could be important. Such a model could help determine how microglial responses in multi-cellular assemblies such as neural synapses differ from *in vitro* preparations. It is important to recognize that for Ca^2+^ responses in cultured or immortalized microglia, doses of 50–100 µM ATP are commonly used (Hide et al., 2000; Barberà-Cremades et al., 2012). In contrast, extracellular ATP concentrations in the brain are on the order of 5–10 nanomolar (Faroqi et al., 2021) under physiological conditions, though insults including moderate traumatic brain injury and ischemia can elevate ATP two- to 4-fold (Melani et al., 2017; Peng et al., 2019; Moro et al., 2021). To reconcile these disparate concentrations, micro-domains formed between ATP-releasing and ATP-sensing cells may locally increase concentrations (Rahmaninejad et al., 2020), similar to locally defined intracellular Na^+^ and Ca^2+^ domains (Shigetomi et al., 2010; Aronsen et al., 2013; Peglow et al., 2013).

### 4.3 Purinoreceptors control microglia migration and displacement

Our study contributes a quantitative model linking *P*2*Y*12 activation to the PI3K and Akt axis essential for microglia migration (Ohsawa et al., 2004; Ohsawa et al., 2007; Irino et al., 2008). Unlike the metabotropic *P*2*Y* receptors implicated in intracellular Ca^2+^ signals, *P*2*Y*12 activates *G*_*i*/*o*_, which stimulates PI3K and ultimately phosphorylation of Akt (Ohsawa et al., 2004). Our model reproduces the rate of PI3K-dependent Akt phosphorylation in addition to migration distances inferred from Ohsawa et al. (2004). Interestingly, both our model and data from (Ohsawa et al., 2004) suggest that Akt phosphorylation is slow and reaches its maximum about 3 minutes after ATP treatment. This contrasts with the rapid onset of migration observed by others (Dou et al., 2012; Etienne et al., 2019). For instance, supplemental movies from Dou et al. (2012) indicate that microglia migrate almost immediately in response to ATP and approach a rate of [1.7 μm/s] within 10 min of 1 mM ATP. Similar findings for microglia in tissue slices were also reported (Etienne et al., 2019). Hence, either low levels of phosphorylated Akt are sufficient for invoking migration at early time points, or alternatively, *P*2*Y*12- or *p*Akt-independent mechanisms mediate the rapid onset of migration. In support of the latter speculation, our model and experiments from Ohsawa et al. (2004) demonstrate that *P*2*Y*12 −/− dramatically reduces, but does not entirely eliminate, migration.

In this regard our model was constructed to reflect the Ca^2+^-dependence of microglial migration, given observations suggesting that 1) *P*2*Y*12 silenced microglia migrate and 2) extracellular Ca^2+^ significantly enhances migration by promoting Akt phosphorylation (Ohsawa et al., 2004; Ohsawa et al., 2007). As such, our simulation recapitulates data indicating that migration is significantly reduced when *P*2*X* contributions are neglected (Ohsawa et al., 2004; Ohsawa et al., 2007). This finding suggests that there are ATP-triggered, Ca^2+^-dependent migration mechanisms (Ohsawa et al., 2004; Ohsawa et al., 2007; Irino et al., 2008) that could be sensitive to rapid Ca^2+^ signals, such as those exhibited by P2X4. These mechanisms could include Ca^2+^-dependent recruitment of PI3K to the plasma membrane (Ohsawa et al., 2004), activation of the Ca^2+^-binding protein Iba (Ito et al., 2001), regulation of cytoskeletal proteins (Li et al., 2009), and regulation of myosin by the CaM-dependent myosin light-chain kinase (Shimizu et al., 2006). To our knowledge, the rates of these mechanisms have not been examined in microglia, which precluded us from explicitly representing these processes in our model. Nonetheless it is plausible that *P*2*X* receptors trigger Ca^2+^-dependent migration machinery that initiate migration, after which the more gradual activation of the PI3K/Akt axis *via* *P*2*Y*12 sustains migration over longer time intervals. Along these lines, low-amplitude Ca^2+^ oscillations from metabotropic *P*2*Y* receptors likely enhance migration, which is consistent with the requirement of *IP*_3_-induced Ca^2+^ release for *P*2*Y*12-driven chemotaxis (Kettenmann et al., 2011). Clearly, ATP-induced migration in microglia is exceedingly complex (reviewed in (Stephens et al., 2008; Cianciulli et al., 2020)) and warrants further investigation to unravel the intricate relationships between Ca^2+^ dynamics and migration.

Our simulations implicate Ca^2+^ signaling in promoting migration as well as TNF*α* synthesis. This raises the question as to whether ATP can stimulate microglia displacement associated with homeostatic functions, such as supporting neuron development and transmission (Schafer et al., 2012), without driving inflammatory cytokine responses. It is apparent from our simulations that a key distinction between these cellular responses is the duration of the intracellular Ca^2+^ waveform. Namely, our simulations show that sub-micromolar ATP treatments yield short-lived Ca^2+^ waveforms ( < 2 min) that are nonetheless sufficient for movement. In contrast, we show that higher amplitude Ca^2+^ waveforms or blocking ectonucleotidase activity prolongs *P*2*Y* and P2X4 Ca^2+^ transients and thereby increases TNF*α* mRNA production (Supplementary Figure S8). Similar prolonged Ca^2+^ signals are associated with inflammatory microglia (Hoffmann et al., 2003; Kettenmann et al., 2011) and are routinely induced *via* millimolar ATP treatment to activate P2X7, or reagents including LPS and ionomycin (Hoffmann et al., 2003). It is apparent that the slow rate of activated transcription factor translocation into the nucleus, which can occur over minutes (Bazzazi et al., 2015), necessitates prolonged Ca^2+^ transients to induce transcription of inflammatory products like TNF*α*. This was reflected in our model for NFAT and was experimentally demonstrated for Ca^2+^ ionophore treated HEK293 cells in (Bazzazi et al., 2015).

### 4.4 Differential purinergic receptor expression and its impact on microglia function

Our model suggests that the expression levels of P2 receptors enable microglia to regulate migration and pro-inflammatory responses to ATP (Lively and Schlichter, 2013). This occurs in part through modulating intracellular Ca^2+^ dynamics. Since *P*2*X* and *P*2*Y* receptors exhibit unique and diverse Ca^2+^ waveforms (Egan and Khakh, 2004), we hypothesized that phenotype-specific differences in P2 receptor expression in microglia influence both 1) Ca^2+^ responses and 2) migration. We investigated this hypothesis by adapting P2 expression levels in the model based on published BV2 cell mRNA data. Model predictions of ATP-stimulated Ca^2+^ waveforms and migration were compared against experiments with BV2 cells.

The mRNA data used for our model (He et al., 2018) showed that primary cells and BV2 microglia had similar numbers of P2X7 and *P*2*Y*2 transcripts, but BV2 cells had fewer P2X4 mRNA transcripts. For simplicity, we assumed that the purinergic receptor activity in our model correlated with mRNA expression. Based on our assumptions the model predicted Ca^2+^ waveforms in BV2 cells that were qualitatively similar to those simulated for primary cells. In contrast, *P*2*Y*12 mRNA transcripts were reduced 30-fold in BV2 cells relative to primary cells (He et al., 2018). Accordingly, our model predicted diminished migration in BV2 cells. We did not observe directed migration in our BV2 cell assays, which is consistent with other reports that confirm negligible or very slow migration responses to chemotactic stimuli (Gyoneva et al., 2009; He et al., 2018; Franco-Bocanegra et al., 2019) Nonetheless, we observed that the BV2 cells exhibited minor membrane movements like ruffling and displacement in response to ATP. The displacements increased in an ATP-dose dependent manner consistent with our model predictions for migration. While data quantifying receptor expression and membrane localization is ultimately needed to better infer receptor activity, our simulations suggest predicting cell function from transcriptomic or proteomics data could become feasible. It should generally be noted that qPCR and Western blot data are obtained from a large ensemble of cells, whereas the Ca^2+^ and membrane displacements we report were measured on a single cell basis. Therefore, we would expect to see greater variability in the behavior of single cells than might be inferred from qPCR alone. Along these lines, in principle, mRNA data from *in situ* microglia could be used to predict the phenotype of tissue microglia. Such mRNA data are available for acutely isolated microglia prior to culturing (Crain et al., 2009) in order to facilitate comparisons of *in situ*-like microglia with primaries. Nonetheless, the tissue environment also contains a variety of molecular factors that will undoubtedly affect microglial phenotypes beyond protein expression alone.

## 5 Limitations

There are several model limitations that can guide refinement in subsequent studies. A prominent limitation is that many of the underlying biological processes linking ATP binding to migration and cytokine responses are not completely resolved. Of those we considered in our model, the kinetics of those processes have not been determined in primary microglia in sufficient detail. The Ca^2+^ responses induced by the purinoreceptors are perhaps the best characterized of these processes, as time-dependent fluorescence data were available. Other processes though were heavily reliant on western blotting and microscopy, which are qualitative in nature and based on pooled cells. Although we limit the scope of our study to the purinoreceptors that respond to ATP and ADP, nucleotidases degrade these nucleotides into adenosine that could activate adenosine receptors in microglia (Merighi et al., 2017; Borea et al., 2018). The activation of the adenosine receptor A2aR, for instance, has been shown to contribute to neuroinflammation (Colella et al., 2018).

We also assumed that the biochemical pathways are spatially homogeneous within the cell for the simplicity of modeling and parameterization. Nonetheless, a number of proteins have precise subcellular localization or undergo changes in a localization about activation, such as P2X4 (Toulme and Khakh, 2012) and NFAT (Bazzazi et al., 2015). Accounting for these changes could impact their ability to promote gene transcription *versus* migration responses.

It was evident from our measurements of ATP-induced Ca^2+^ waveforms that cell-to-cell variation in responses was substantial. Namely, many of the individual cells presented traces that strongly deviated from the mean (see Figure 7). Our model aims to qualitatively capture calcium transients following ATP stimulation. Data from Ikeda et al. represents the ensemble average over many cells, therefore, exact (quantitative) matches are generally not feasible owing to the stochastic nature of Ca^2+^ transients. However, our data captures the qualitative trends of elevated Ca^2+^ immediately after ATP addition that dissipates with time. Our modeling approach relies on deterministic equations, which are most appropriate for describing the average behavior of a large ensemble of cells. This approach is valid, given that many of the experimental data used to train our approach were from western blots and mRNA quantification, which generally use large pools of cells. Stochastic models such as that from Skupin et al. (2008); Skupin et al. (2010); Cao et al. (2013) could be used in complement to our model to investigate how cell-to-cell variations in gene expression or protein activity could impact ATP-induced Ca^2+^ waveforms. At the very least, the cell-to-cell variability underscores a need for single-cell characterization of cell genotypes and phenotypes, as well as sensitivity analyses such as in Figure 6 to better characterize Ca^2+^ waveforms and their effects in diverse microglial cell populations. Metabotropic purinoreceptors in principle can also stimulate the production of cyclic AMP (cAMP). We did not investigate dependencies of migration or TNF*α* production on cAMP production in principle *P*2*Y*11 activation promotes Gs signaling, which would result in cAMP production (Erb and Weisman, 2012). However, *P*2*Y*11 receptors do not appear to be significantly expressed in murine primary and BV-2 microglia (Brautigam et al., 2005). Despite this, it has been reported that TNF*α* production reduces cAMP (Patrizio, 2004), cAMP signaling can inhibit microglia migration (Umpierre and Wu, 2021), and that *P*2*Y*12 activation blocks cAMP synthesis *via* Gi (Tozaki-Saitoh et al., 2017). Future investigations of other GPCRs that utilize Gs signaling would be helpful to better understand how other messengers, such as cAMP, contribute to migration and cytokine production.

Our model could be improved by accounting for *K* ^+^-dependent signaling. There are a multitude of mechanisms by which changes in intracellular *K* ^+^ and membrane potential could influence microglial signal transduction, such as by enhancing the electromotive force for Ca^2+^ entry, or by influencing the activity of the sodium/*K* ^+^ ATPase. Reflecting these contributions may provide more complete descriptions of *K* ^+^ mechanisms mediating inflammation (Stebbing et al., 2015) and migration (Swiatkowski et al., 2016). For instance, *P*2*Y*12-dependent activation of *K* ^+^ channel dynamics is believed to contribute to migration (Kettenmann et al., 2011). This is supported by studies suggesting that *P*2*Y*12-activation induces substantial outward current associated with *K* ^+^ channel activity (Swiatkowski et al., 2016; Fan et al., 2017). It is further understood that *K* ^+^ efflux constitutes an important stage of priming the microglial inflammasome, which is necessary for maturating pro-inflammatory cytokines such as IL-1*β* (Xu et al., 2020). Along these lines, it is increasingly recognized that *P*2*X*7 and P2X4 conduct *K* ^+^ countercurrent when activated (Yaron et al., 2015; Nguyen et al., 2020); moreover, changes in *K* ^+^-channel expression upon microglial activation may contribute to these responses (Nguyen et al., 2017).

While we modeled the PI3K/*p*Akt axis, RAGE/RhoA/ROCK are also involved in mediating chemotaxis (Tatsumi et al., 2015; Zhang et al., 2019). For instance, it was shown that inhibition of ROCK *via* H-1152 reduced p38 phosphorylation and membrane ruffling following ATP-dependent *P*2*Y*12/13 activation (Tatsumi et al., 2015). Inhibition of MLCK, Rac1 and p38 also results in the attenuation of migration in primary cultured murine microglia (Miller and Stella, 2009). p38 is also known to be Ca^2+^ sensitive (Trang et al., 2009), which again introduces another potential pathway sensitive to Ca^2+^ waveform characteristics. Related to this, CaM dependent contributions to migration are also important. O’Brien *et al* found that active CaM was involved in chemotaxis by monitoring the activity of its phosphodiesterase (PDE1) target (O’Brien et al., 2020). Similarly, Yao *et al* demonstrated the involvement of CaM in migration *via* CaM-dependent MLCK (Yao et al., 2013). *P*2*Y*2 -mediated epidermal growth factor receptor (EGFR) is also an established pathway contributing to cell migration and cytokine production (Li et al., 2015). Altogether, the dependencies of cell migration on diverse signaling pathways suggest microglia are highly adaptive to a variety of extracellular stimuli to promote migration responses. Hence, resolving these inter-dependencies will warrant additional studies and their dependencies on Ca^2+^ to delineate microglial migration responses specific to ATP.

## 6 Conclusion

ATP-induced Ca^2+^ waveforms in microglia have diverse properties, such as amplitude, duration and oscillatory behavior. These properties depend on which P2 receptor types are activated, in addition to ectonucleotidase activity. In this study, we developed a computational model to predict how P2 receptors and ectonucleotidase hydrolases control Ca^2+^ waveforms in microglia that in turn influence microglia migration and cytokine production. With this model, we examine the propensity for these diverse Ca^2+^ waveforms to drive these canonical microglial responses to ATP.

We interpret these results in light of our previously published microglia model for probing *P*2*X* contributions to TNF*α* mRNA synthesis as a model for pro-inflammatory cytokine responses to ATP (Chun et al., 2019). In that study, we demonstrated that Ca^2+^ waveforms generated by *P*2*X* activation are typically high-amplitude and of finite duration. With the addition of *P*2*Y*, we predict a wider diversity of Ca^2+^ waveforms that can include stable and damped oscillations of low amplitude and frequency. Interestingly, our modeling results highlight a complementary role of ectonucleotidase activity, namely CD39, in controlling the ATP pool available to trigger such responses, by hydrolyzing ATP and ADP into AMP. This finding is particularly important since CD39 and its complementary CD73 ectonucleotidase, which hydrolyzes AMP, undergo significant expression changes when microglia are activated (Jakovljevic et al., 2019).

Our simulations indicate that the distinct Ca^2+^ waveforms shaped by P2 receptors and CD39 selectivity control downfield signaling pathways. We investigated this selective control by simulating migration *versus* cytokine production responses as a function of the Ca^2+^ waveforms generated by P2 receptors. Our model indicates that short-duration, oscillatory Ca^2+^ transients induced by *P*2*Y* receptors and P2X4 with micromolar ATP are sufficient to promote migration responses without significantly inducing TNF*α* production. On the other hand, millimolar ATP concentrations that activate *P*2*X*7 supported sustained cytosolic Ca^2+^ levels that could trigger TNF*α* release. In a similar vein, we can expect responses would vary with the types or combinations of nucleotides used to stimulate purinoreceptors. We demonstrate this as a proof-of-principle for UTP in Supplementary Figure S9. In this case, UTP selectively activates *P*2*Y*2 receptors (Sophocleous et al., 2020) and *P*2*Y*6 receptors (Jiang et al., 2017), while ionotropic P2 receptors are unresponsive to UTP (Visentin et al., 1999; Kanellopoulos et al., 2021). This resulted in moderate Ca^2+^ transients with spikes from IP3R activation, but lacked the high-amplitude fluctuations associated with *P*2*X* receptors; we anticipate that such transients would not be expected to induce significant TNF*α* production. Overall, these findings illustrate how microglia orchestrate complex cytokine and migration functions in response to ATP as well as other damage associated with molecular patterns like UTP (Cieślak et al., 2011).

The concerted roles of *P*2*X*, *P*2*Y*, and ectonucleotidase proteins in mediating cellular responses to ATP further suggest how changes in gene expression shape microglia responses to stimuli. For instance, higher *P*2*Y*12 expression in resting relative to pro-inflammatory microglia likely favor migration responses to ATP in the former. Similarly, elevated P2X4 and P2X7 expression in pro-inflammatory microglia sensitize cytokine responses to ATP. These differences in microglia responses following changes in gene expression underscore the need for models to account for changes in protein activity.

Robust characterization of detailed signaling networks in diverse microglia phenotypes remains a significant challenge. This is especially challenging for tissue resident microglia that are difficult to experimentally manipulate *in situ*. For this reason, we tested if our model could leverage mRNA transcript data from the BV2 microglial cell line to approximate changes in P2 receptor activity. Using those mRNA data, we found that the model predicted Ca^2+^ waveforms and migration responses to ATP that were reasonably consistent with experiments we conducted using the BV2 microglia cell line. This raises the possibility that coupling models trained from cultured primary or immortalized cells *in vitro* with transcriptomic and proteomic data could enable predictions of cellular responses from *in vivo* or disease-associated microglia, for which extensive functional testing is not feasible. Related to this, since dysfunctional microglial responses are associated with neurological disorders including chronic pain, Alzheimer’s Disease and Parkinson’s Diseases (GBD 2017 US Neurological Disorders Collaborators, 2021), our computational model may be an invaluable tool to probe mechanisms underlying these diseases. Toward this end, future experiments could investigate model predictions, such as assessing how partial CD39-inhibition influences microglia migration and cytokine production. Similarly, quantitative experimental characterization of other microglial processes dependent on Akt and Ca^2+^ could be helpful, such as cytoskeletal rearrangement (Nishimoto et al., 2021), phagocytosis (Olmedillas Del Moral et al., 2019), and autophagy (Jiang et al., 2019; Desale et al., 2021).

## Data Availability

The data analyzed in this study was obtained from https://github.com/huskeypm/pkh-lab-analyses/tree/master/2021-P2Y-microglia. Requests to access these datasets should be directed to the corresponding authors.
