# Supplementary material for: Purinoreceptors and ectonucleotidases control ATP-induced calcium waveforms and calcium-dependent responses in microglia: Roles of P2 receptors and CD39 in ATP-stimulated microglia
Source: Front Physiol. 2023 Jan 9;13:1037417. doi: 10.3389/fphys.2022.1037417 (PMC9868579; doi:10.3389/fphys.2022.1037417)
Supplement: Supplementary file 1 [file DataSheet1.pdf]

S Supplement

S.1 Pathway Summary and References

Table S1: Reactions used in the computational microglia model

Rxn No.	Rxn	Cell	Reference
1	P2Y $\Rightarrow IP_3 \Rightarrow$ [Ca^{2+}]$_i$	heptaocyte	[6, 7]
2	$P2X4 \Rightarrow$ [Ca^{2+}]$_i$	microglia	[28, 10]
3	$P2X7 \Rightarrow$ [Ca^{2+}]$_i$	microglia	[16, 3]
4	[Ca^{2+}]$_e \Rightarrow$ [Ca^{2+}]$_i$ (leak)		Fit
5	NCX $\Rightarrow$ [Ca^{2+}]$_e$	microglia	[25]
6	[Ca^{2+}]$_i \Rightarrow$ Buffers		Fit
7	[Ca^{2+}]$_i \Rightarrow$ CaM $\Rightarrow$ CN	Cardiac	[11, 2]
8	[Ca^{2+}]$_i \Rightarrow$ SERCA	Cardiac	[25]
9	[Ca^{2+}]$_{ER} \Rightarrow$ [Ca^{2+}]$_i$ (leak)		Fit
10	[Ca^{2+}]$_{ER} \Rightarrow$ Calreticulin (Calsequestrin)	astrocytes	[25]
11a	[Ca^{2+}]$_i \Rightarrow$ p-p38	microglia	[29]
11b	p-p38 $\Rightarrow$ TNFα	microglia	[13]
12a	CN $\Rightarrow$ NFAT	microglia	[32, 5]
12b	NFAT cycle	myocyte	[5]
12c	NFAT $\Rightarrow$ TNFα	microglia	[20]
13	TNFα Production	microglia	Fit
14	Activation of $P2X7 \Rightarrow$ TNFα release	microglia	[1]
15	$P2Y12 \Rightarrow G_{i/o}$	microglia	[15, 23]
16,17	$G_{i/o} \Rightarrow$ PI3K $\Rightarrow$ pAkt	microglia	[23, 8]
18	pAkt + [Ca^{2+}]$_i \Rightarrow$ Migration	microglia	[23]
19	NTPDase of ATP	COS-7	[18, 24]

S.2 Mathematical Models and Parameters

Table S2 displays the physical constants used for the model.

Table S2: General Parameters

Parameters	Values	Units
T	310	K
V_{pot}	-5.0×10^{-2}	V
F	96485	$\frac{C}{mol}$
R	8.314	$\frac{J}{mol \times K}$
$r_{microglia}$	3.68	μm
$[Ca^{2+}]_e$	2	mM
$[Na^{+}]_e$	145	mM
$[Na^{+}]_i$	8	mM

S.2.1 GPCR-mediated Ca^{2+} transients

Equations Our model is based on the formulations from Cuthbertson-Chay and Young-Kaizer [6, 7]. The Cuthbertson-Chay model (denoted as CC) describes the G-protein coupled receptor signaling pathway culminating in the production of † and IP_3 from PIP2. The Young-Kaizer model (denoted as YK) describes IP_3-dependent Ca^{2+} release from the ER via the . The CC model equations are as follows:

$$\begin{aligned}
d_{cc} &= \frac{[DAG]_{cc}}{K_{d,cc} - \frac{0.1}{1+(\frac{kd_{3,cc}}{[ATP]})^5}} \\
d*_{cc} &= \frac{[DAG]_{cc}}{K_{d,cc}} \\
g_{cc} &= \frac{[G_\alpha - GTP]_{cc}}{K_{g,cc}} \\
R_{PKC,cc} &= \frac{[DAG]_{cc}[Ca^{2+}]_i}{K_{p,cc}(K_{c,cc} + [Ca^{2+}]_i)} \\
r_{cc} &= \frac{R_{PKC,cc}}{K_{R,cc}} \\
R_{PLC,cc} &= \frac{(d*_{cc})^{n_{cc}}(g_{cc})^{m_{cc}}}{(1 + (d*_{cc})^{n_{cc}})(1 + (g_{cc})^{m_{cc}})(1 + (r_{cc}^{o_{cc}})}
\end{aligned} \tag{S1}$$

These expressions dictate the DAG, ATP, and Ca2+ dependent activities of PKC and PLC. The d terms reflect ATP-dependent and spontaneous (ATP-independent) activation of PLC to capture Ca^{2+} transients triggered by P2Y receptor activation versus rest conditions, respectively, as we will describe further below.

These terms are then used to evaluate state models for the GTP-bound Ga protein, activate P2Y receptors, and DAG/IP3 as follows:

$$
\begin{aligned}
[IP_3]_{cc} &= [DAG]_{cc} \\
\frac{d[G_\alpha - GTP]_{cc}}{dt} &= k_{g,cc}\frac{[ATP]*_{cc}[P2Y]_{cc}}{k_{a,cc}+[ATP]*_{cc}}[P2Y]_{cc}([G_{\alpha,total}]_{cc} - [G_\alpha - GTP]_{cc}) \\
&\quad - h_{g,cc}[G_\alpha - GTP]_{cc} \\
\frac{d[P2Y]_{cc}}{dt} &= k_{P2Y,cc}([P2Y]_{total,cc} - [P2Y]_{cc}) - h_{P2Y,cc}[P2Y]_{cc}R_{PKC,cc} \\
\frac{d[DAG]_{cc}}{dt} &= k_{d,cc}R_{PLC,cc} - h_{d,cc}[DAG]_{cc} + \gamma[ATP]*_{cc}
\end{aligned}
\tag{S2}
$$

Eqns S2 are called twice per time-step in the model, to reflect the different values of $R_{PLC,cc}$ computed for ATP-dependent and spontaneous conditions, as well as 'activity' terms used for determining activated G_α:

$$
\begin{aligned}
[ATP]_{cc} &= \frac{0.010}{(1+\frac{kd_{1,cc}}{[ATP]})} + \frac{0.010}{(1+\frac{kd_{2,cc}}{[UTP]})} \\
[ATP]*_{cc} &= 0.010
\end{aligned}
\tag{S3}
$$

The first expression reflects that ATP and UTP can be used to trigger the P2Y receptors that are commonly expressed in microglia, e.g. P2Y2 and P2Y6 [12, 15] (see main text for further elaboration). The second expression is chosen to be a constant in order reflect ATP-independent activity at resting conditions that culminates in spontaneous Ca^{2+} release from s [30]. We also utilized the Ca^{2+}-dependent model for PKC activation from [6], though some PKC isoforms may be activated in a Ca^{2+}-independent fashion [19] Lastly, the original CC model reflects PKC-dependent inhibition of $G_{\alpha q}$, whereas more recent studies instead implicate PKC inhibition of PLC-β [19]. Accordingly, we removed the PKC-dependent $G_{\alpha q}$ inhibition step in favor of PKC/PLC-β inhibition (Eqn S1). PLC-β activity may also be influenced by Ca^{2+}[19], but we omitted this potential dependence owing to the lack of data to describe the dependency. Corresponding parameters for these equations are provided in Table S3.

-mediated Ca^{2+} release from the ER is described by the following equations from :

Table S3: Parameters associated with the CC model listed in Eqn S3-S2. Values in the parentheses reflect activity at resting (ATP-independent) conditions.

Parameters	Values	Units
$K_{g,cc}$	51	nM
$K_{d,cc}$	4.4 (2.9)	nM
$K_{s,cc}$	5	nM
$K_{p,cc}$	40	nM
$K_{c,cc}$	20	nM
$k_{g,cc}$	20	1/s
$k_{c,cc}$	700	nM/s
$h_{c,cc}$	0.4	1/s
$k_{d,cc}$	31	nM/s
$k_{d1,cc}$	20	µM
$k_{d2,cc}$	10	µM
$k_{d3,cc}$	170	µM
$h_{d,cc}$	0.62	1/s
$k_{a,cc}$	10	µM
$k_{P2Y,cc}$	0.01	1/s
$h_{P2Y,cc}$	10	1/s
n_{cc}	2	
m_{cc}	4	
o_{cc}	1	
$K_{R,cc}$	0.5	
$r_{g,cc}$	1.0	nM/s
$h_{g,cc}$	5.0	1/s
$[G_\alpha]_{total}$	150	nM
$[P2Y]_{total}$	150	nM
γ	10	

$$
\begin{aligned}
e_{1,yk} &= 1 + \frac{[IP_3]_{yk}}{d_{1,yk}} \\
e_{2,yk} &= 1 + \frac{[IP_3]_{yk}}{d_{3,yk}} \\
x_{011,yk} &= \frac{1 - (x_{000,yk}e_{1,yk} + x_{010,yk}e_{1,yk} + x_{001,yk}e_{3,yk})}{e_{3,yk}} \\
x_{110,yk} &= \frac{x_{010,yk}[IP_3]_{yk}}{d_{1,yk}} \\
x_{111,yk} &= \frac{x_{011,yk}[IP_3]_{yk}}{d_{3,yk}} \\
x_{100,yk} &= \frac{x_{000,yk}[IP_3]_{yk}}{d_{1,yk}} \\
x_{101,yk} &= \frac{x_{001,yk}[IP_3]_{yk}}{d_{3,yk}} \\
V_{1,yk} &= a_{4,yk}([Ca^{2+}]_{i,yk}x_{000,yk} - d_{4,yk}x_{001,yk}) \\
V_{2,yk} &= a_{4,yk}([Ca^{2+}]_{i,yk}x_{010,yk} - d_{4,yk}x_{011,yk}) \\
V_{3,yk} &= a_{5,yk}([Ca^{2+}]_{i,yk}x_{000,yk} - d_{5,yk}x_{010,yk}) \\
V_{4,yk} &= a_{5,yk}([Ca^{2+}]_{i,yk}x_{001,yk} - d_{5,yk}x_{011,yk}) \\
J_{1,yk} &= c_{1,yk}(v_{1,yk}x^3_{110,yk} + v_{2,yk})([Ca^{2+}]_{ER,yk} - [Ca^{2+}]_{i,yk}) \\
J_{2,yk} &= \frac{v_{3,yk}[Ca^{2+}]_{i,yk}}{[Ca^{2+}]^2_{i,yk} + k^2_{3,yk}} \\
\frac{dx_{000,yk}}{dt} &= -V_{1,yk} - V_{3,yk} \\
\frac{dx_{001,yk}}{dt} &= V_{1,yk} - V_{4,yk} \\
\frac{dx_{010,yk}}{dt} &= V_{3,yk} - V_{2,yk} \\
J_{IP_3R} &= (J_{1,yk} - J_{2,yk})f_{conversion}
\end{aligned}
\tag{S4}
$$

The x terms represent states of the involved in the channel's gating, such as those activating ER Ca^{2+} release (see $J_{1,yk}$). The V terms help determine the Ca^{2+}-dependent inactivation of the (see $J_{2,yk}$), which results in a negative feedback inhibition motif that gives rise to Ca^{2+} oscillations. The net Ca^{2+} released by the , J_{IP_3R} reflects the competing activation and inactivation terms. Parameters used in these equations are given in Table S4.

This model assumes Ca^{2+} release as a deterministic process, though the Ca^{2+} transients resemble stochastic spiking in experiment [26]. We note that the parameters provided for the respective CC and YK models used different units (e.g. uM vs nM), therefore conversion factors were used to align the units.

The corresponding sets of equations are now In this model, there are two sets of equations to reproduce the Ca^{2+} transients mediated by the IP_3R activity.

The first set of the Cutherbertson-Chay and Young-Kaizer model equation

Table S4: Parameters associated with S3. Values in the parentheses reflect activity at resting (ATP-independent) conditions.

Parameters	Values	Units
$c_{0,yk}$	2.0	µM
$c_{1,yk}$	0.185	
$v_{1,yk}$	2.0×10^3 (100)	1/s
$v_{2,yk}$	0.11	1/s
$v_{3,yk}$	0.9	1/(µM-s)
$k_{3,yk}$	0.1	µM
$k_{4,yk}$	1.1	µM
$a_{1,yk}$	4.0×10^2	1/(µM-s)
$a_{2,yk}$	0.2	1/(µM-s)
$a_{3,yk}$	4.0×10^2	1/(µM-s)
$a_{4,yk}$	0.2	1/(µM-s)
$a_{5,yk}$	20	1/(µM-s)
$d_{1,yk}$	3.0×10^{-3}	µM
$d_{3,yk}$	6.0×10^{-3}	µM
$d_{4,yk}$	0.4	µM
$d_{5,yk}$	5.0×10^{-3}	µM
$f_{conversion}$	30 (200)	nM/µM

is less ATP sensitive and results in larger spikes as the receptor is activated by IP3. The second set of the model equations generates ATP-independent Ca^{2+} oscillations[30], the amplitude of which is smaller than the oscillations generated by the ATP-dependent model (the first set).

Although there is no direct evidence that IP_3 maintains the Ca^{2+} baseline transients, it is fair to say that the IP_3-mediated pathway is not amplified rather than terminated or silenced due to the absence of ATP. Therefore, instead of developing a new set of mathematical expressions to mimic spontaneous Ca^{2+} baseline, we again adapted the CC and YK integrated model with slightly tuned parameters to mimic the randomness of the baseline transients but the mathematical expression remains a deterministic model.

The input and fitted parameters are reflected in Tables S3 and S4) to best reproduce experimentally-measured intracellular Ca^{2+} transients in microglia.

The difference between the ATP-dependent and independent equations are marked by asterisk (*) in Eqn S3. The $[ATP]_{cc}$ in the ATP-dependent mechanism is the trigger for the subsequent pathways, whose magnitude is based on both the concentration of ATP and UTP, since the majority of $P2Y$ receptor consists of P2Y2 and P2Y6 [12, 15]. On the other hand, the ATP-independent mechanism is simply expressed as a constant variable that triggers consistent oscillatory waveform of Ca^{2+} in the system to mimic the baseline in microglia [30]. Unless it is listed, the ATP-independent Ca^{2+} baseline transients were generated by the identical model with the set of adjusted parameters listed in Table S3 and S4.

The YK model also includes feedback inhibition of IP3R by Ca^{2+}[31]. Although PLC-β is calcium-dependent, it is omitted in the model for simplicity [19].

$$
\begin{aligned}
[IP_3]_{yk} &= [IP_3]_{cc}/1000 \quad (nM \rightarrow \mu M) \\
[Ca^{2+}]_{i,yk} &= [Ca^{2+}]_i/1000 \quad (nM \rightarrow \mu M) \\
[Ca^{2+}]_{ER,yk} &= [Ca^{2+}]_{ER}/1000 \quad (nM \rightarrow \mu M)
\end{aligned} \tag{S5}
$$

To integrate two models, the calculation performs the unit conversion for $[IP_3], [Ca^{2+}]_i$ and $[Ca^{2+}]_{ER}$ as shown Eqn S5.

S.2.2 Markov State Modeling based P2X receptor kinetic model

The following parameters were refit from [4] (P2X-only) to reflect simultaneous contributions from P2X and P2Y-class receptors.

$P2X4$ The mathematical expression is available in the previous work [4].

Table S5: Parameters associated with $P2X4$ receptor kinetics

Parameters	Values	Units
k_1	1.0	1/s
k_2	2.61×10^5	1/(M×s)
k_3	0.1	1/s
k_4	1.6×10^5	1/(M×s)
k_5	0.25	1/s
k_6	8.0×10^6	1/(M×s)
H_1	0.02	1/s
H_2	0.0	1/s
H_6	0.1	1/s

$P2X7$ Equations describing $P2X7$ activation are described below:

$$
\begin{aligned}
s &= \frac{1}{1+(\frac{k_d}{[ATP]})^n} \\
k_4 &= k_{4,low} - s(k_{4,low} - k_{4,high}) \\
k_6 &= k_{6,low} - s(k_{6,low} - k_{6,high}) \\
h_7 &= H_{7,low} + s(H_{7,high} - H_{7,low}) \\
h_{1,p} &= 2k_4[ATP] \\
h_{1,m} &= 2k_3 \\
h_{2,p} &= k_6[ATP] \\
h_{2,m} &= 3k_5 \\
H_2 &= \frac{h_{2,p}}{h_{2,m}} \\
y_{1,p} &= h_{1,p} \\
y_{1,m} &= \frac{h_{1,m}}{1+H_2} \\
\\
D_1 &= 1 - (D_2 + D_{34} + C_1 + C_2 + Q_{12}) \\
\frac{dD_2}{dt} &= 3k_2[ATP]D_1 + H_2C_2 + y_{1,m}D_{34} - (k_1 + y_{1,p} + H_7)D_2 \\
\frac{dD_{34}}{dt} &= y_{1,p}D_2 - y_{1,m}D_{34} \\
\frac{dC_1}{dt} &= H_1D_1 + k_1C2 - 3k_2[ATP]C_1 \\
\frac{dC_2}{dt} &= H_7D_2 + 3k_2[ATP]C_1 + y_{1,m}Q_{12} - (k_1 + y_{1,p} + H_2)C_2 \\
\frac{dQ_{12}}{dt} &= y_{1,p}C_2 - y_{1,m}Q_{12}
\end{aligned}
\tag{S6}
$$

Table S6: Parameters associated with $P2X7$ receptor kinetics from Eqn S6. An ATP-dependent scaling term s is introduced to limit $P2X7$ activation for low micromolar ATP concentrations that tend to exclusively activate $P2X4$ receptors [28, 17, 3, 4].

Parameters	Values	Units
k_1	190	1/s
k_2	8.13×10^3	1/(M×s)
k_3	0.04	1/s
k_5	0.07	1/s
H_1	5.0×10^{-3}	1/s
H_2	0.3	1/s
H_5	0.0	1/s
H_6	0.0	1/s
$k_{4,low}$	1.0×10^2	1/(M×s)
$k_{6,low}$	5.0×10^2	1/(M×s)
$H_{7,low}$	1.0×10^3	1/s
$k_{4,high}$	7.0×10^3	1/(M×s)
$k_{6,high}$	0.1	1/(M×s)
$H_{7,high}$	0.008	1/s
k_d	420	μM
n	15	

S.2.3 Homeostasis Equations in Microglia

Ca^{2+}-handling in the ER lumen ($[Ca^{2+}]_{ER}$) Our model for Ca^{2+} handling in the ER(Eqn S7) reflects of the Ca^{2+} release via SERCA ($J_{ERtoCyt,SERCA}$), buffer by calsequestrin or other ER-resident Ca^{2+} binding proteins ($R_{Ca^{2+}\cdot S}$), and Ca^{2+} release via IP_3receptors ($J_{IP_3R,ATP-dependent}$ and $J_{IP_3R,ATP-independent}$). The term denoted 'ATP-independent' represents baseline activity for resting microglia. The leak term ($J_{ERtoCy,Leak}$) represents residual Ca^{2+} from the ER to offset SERCA Ca^{2+} uptake. Detailed expressions for these contributions are found in our previous work [4].

$$\begin{aligned}\frac{d[Ca^{2+}]_{ER}}{dt} &= J_{ERtoCyt,SERCA} - R_{Ca^{2+}\cdot S} - J_{ERtoCy,Leak}\frac{V_{microglia}}{V_{ER}} \\ &- J_{IP_3R,ATP-dependent} - J_{IP_3R,ATP-independent}\end{aligned} \tag{S7}$$

Homeostasis in Cytoplasm domain ($[Ca^{2+}]_i$) Our model for Ca^{2+} handling in the cytoplasm domain (Eqn S8) consists of Ca^{2+} uptake via SERCA ($J_{CytoER,SERCA}$), buffering by the calmodulin-calcineurin complex ($R_{Ca\cdot F}$,$R_{Ca\cdot B}$, and $R_{Ca\cdot CaM-CN}$), and Ca^{2+} release via IP_3receptors ($J_{IP_3R,ATP-dependent}$ and $J_{IP_3R,ATP-independent}$). Plasma membrane contributions to the cytosolic Ca^{2+} include the sodium/calcium exchanger (NCX) activity (J_{NCX}) and P2X receptors (J_{P2X7}+J_{P2X4}) introduced in [4]. The leak terms ($J_{ERtoCy,Leak}$ and $J_{ExtoCy,Leak}$) ensure the system remains in steady state at rest. Detailed expressions for these contributions are found in our previous work [4].

$$\begin{aligned}\frac{d[Ca^{2+}]_i}{dt} &= J_{P2X7} + J_{P2X4} + J_{ExtoCy,Leak} + J_{NCX} + J_{ERtoCy,Leak} \\ &+ J_{IP_3R,ATP-dependent} + J_{IP_3R,ATP-independent} \\ &- (J_{CytoER,SERCA} + R_{Ca^{2+}\cdot F} + R_{Ca^{2+}\cdot B} \\ &+ R_{Ca^{2+}\cdot CaM-CN})\end{aligned} \tag{S8}$$

Parameters for our model are provided in Tables S7-S10.

Table S7: Parameters associated with inward current from P2X receptors (equations associated with this set of parameters are listed in [4])

Parameters	Values	Units
$G_{12,P2X4}$	8.15×10^{-10}	$\frac{C}{s \times V}$
$G_{12,P2X7}$	2.0×10^{-8}	$\frac{C}{s \times V}$
$E_{12,P2X4}$	0.0	V
$E_{12,P2X7}$	0.0	V
$f_{I_{Ca^{2+},P2X4}}$	0.0824	
$f_{I_{Ca^{2+},P2X7}}$	0.1	
$f_{conv.,P2X4}$	11	
$f_{conv.,P2X7}$	1	

Table S8: Parameters for NCX activity. The corresponding equations were adapted from Shannon-Bers model [25] and listed in the previous work [4]

Parameters	Values	Units
Q_{10}	1.20	
Kd_{Act}	40.0	nM
n_H	3.44	
H_{Na}	3.60	
V_{max}	35	A/F
η	0.70	
k_{sat}	0.04	
$K_{max,[Ca^{2+}]_i}$	3.63×10^3	nM
$K_{max,[Na^{+}]_i}$	1.23×10^7	nM
$K_{max,[Na^{+}]_e}$	8.75×10^7	nM
$K_{max,[Ca^{2+}]_e}$	1.30×10^6	nM
C_{mem}	1.2×10^{-11}	F

Table S9: Parameters for SERCA activity. The corresponding equations were adapted from Shannon-Bers model [25] and listed in the previous work [4]

Parameters	Values	Units
Q_{10}	2.6	
V_{max}	9.09×10^6	nM/s
K_f	2.80×10^2	nM
K_r	2.10×10^6	nM
H	1.787	

Table S10: Parameters for CaM-dependent activation of calcineurin. The corresponding equations were adapted from [2, 11] and listed in the previous work [4].

Parameters	Values	Units
k_{ab}	1.0×10^{-5}	$1/(nM^2 \times s)$
k_{ba}	10.0	1/s
k_{bc}	1.0×10^{-4}	$1/(nM^2 \times s)$
k_{cb}	1.0×10^3	1/s
$k_{on,A}$	1.0×10^{-2}	$1/(nM \times s)$
$k_{off,A}$	1.0	1/s
$k_{on,B}$	2.0×10^{-6}	$1/(nM^2 \times s)$
$k_{off,B}$	1.0	1/s
$[CaM]_{total}$	100	nM
$[CN]_{total}$	67	nM

Table S11: Parameters Ca^{2+} buffer interaction calculations. The equations associated with the rest of the buffers were adapted from [25] and listed in the previous work [4].

Parameters	Values	Units
$B_{max,F}$	2.5×10^4	nM
$k_{on,F}$	0.15	1/(nM×s)
$k_{off,F}$	23.0	1/s
$B_{max,Br}$	1.0×10^4	nM
$k_{on,B}$	1.0	1/(nM×s)
$k_{off,B}$	1.0×10^3	1/s
$B_{max,S}$	1.4×10^5	nM
$k_{on,S}$	0.1	1/(nM×s)
$k_{off,S}$	6.5×10^4	1/s

S.2.4 Miscellaneous signal transduction

NFAT The NFAT model used in our study was adapted from Cooling *et al*[5] and implemented to link NFAT activation to TNFα synthesis [33]. The equations listed in the previous work [4] were utilized with the parameters (Table S12) fitted in this study.

Table S12: Parameters for NFAT cycle calculations. The corresponding equations were adapted from [5] and listed in the previous work [4].

Parameters	Values	Units
$k_{f,1}$	9.79×10^{-7}	$1/(nM \times s)$
$k_{r,1}$	1.93×10^{-2}	$1/s$
$k_{f,2}$	7.00×10^{-3}	$1/s$
$k_{f,3}$	3.62×10^{-4}	$1/s$
$k_{r,3}$	4.71×10^{-5}	$1/(nM \times s)$
$k_{f,4}$	1.00×10^{-4}	$1/s$
C_{cn}	10	
$[NFAT]_{total}$	1.2	nM

p-p38 The phosphorylation of p38 was simulated by the mathematical model implemented in the previous work [4] to relate phosphorylated p38 to TNFα synthesis.

S.2.5 TNFα

According to a report from Barbera *et al*[1], matured TNFα is present inside the cell and can be exocytosed following the activation of ionotropic P2 receptors. Our model for this process is provide in Eqn S9.

Table S13: Parameters for phosphorylation of p38 calculations. The equations associated with p38 were adapted from [29] and listed in the previous work [4].

Parameters	Values	Units
$[pp38]_{total}$	100	
$k_{b,pp38}$	8.51×10^{-4}	1/s
$k_{f,pp38}$	1.1×10^{-2}	1/s
$k_{d,pp38}$	150	nM
n_{pp38}	5	

$$\begin{aligned}\frac{d[TNF\alpha]_c}{dt} &= D_{nc}([TNF\alpha] - [TNF\alpha]_c) \\ &- ([TNF\alpha]_c - [TNF\alpha]_e)\left(\frac{D_{exo}}{1 + (\frac{k_d}{([Ca^{2+}]_i - [Ca^{2+}]_o})}\right) \\ \frac{d[TNF\alpha]_e}{dt} &= ([TNF\alpha]_c - [TNF\alpha]_e)\left(\frac{D_{exo}}{1 + (\frac{k_d}{([Ca^{2+}]_i - [Ca^{2+}]_o})}\right)\end{aligned} \tag{S9}$$

Table S14: Parameters for simulating TNFα synthesis [4] and the additional equations for the exocytosis of TNFα listed in Eqn S9

Parameters	Values	Units
$k_{trnscrpt}$	2.78×10^{-4}	1/s
k_{trnsl}	2.0×10^{-4}	1/s
$k_{deg,TNF\alpha}$	1.38×10^{-2}	1/s
$k_{deg,mRNA}$	1.35×10^{-4}	1/s
$IC50_1$	0.4	
n_1	2	
$IC50_2$	75.0	
n_2	5.5	
$k_{exp,f}$	5.11×10^{-4}	1/(molecule×s)
$k_{exp,r}$	1.78×10^{-4}	1/s
D_{nc}	10.0	1/s
D_{exo}	5.0	1/s
k_d	25.0	nM
$[Ca^{2+}]_o$	100.0	nM

S.2.6 $P2Y12$-mediated Signaling and Chemotaxis

Ohsawa *et al* [22, 23] determined that $P2Y12$ activation drives migration following ADP treatment (a product of ATP degradation by CD39). [15, 21]. Their latter work determined the augmentation of migration via ionotropic P2 receptor stimulation that promotes Ca^{2+} entry into the cell. Our model for chemotaxis includes both $P2Y12$ activation and the elevation of intracellular Ca^{2+} from $P2X4$ and $P2X7$ receptors (Eqn S10).

$$\begin{aligned}\frac{d[Signal_{Ca^{2+}}]}{dt} &= k_{f,6}([Ca^{2+}]_i - [Ca^{2+}]_o) - k_{b,6}[Signal_{Ca^{2+}}] \\ \frac{d[P2Y12]_{act}}{dt} &= k_{f,1}(f_{P2Y12}([P2Y12]_{total}\rho_{P2Y12} - [P2Y12]_{act}) \\ &\quad + f_{Ca^{2+}}Signal_{Ca^{2+}})[ADP] - k_{b,1}[P2Y12]_{act}\end{aligned} \tag{S10}$$

As shown by Ohsawa *et al*[22, 23], PI3K activation and Akt phosphorylation are proportional to $P2Y12$ activity. This process proceeds through the activation of Gi/o. (Eqn S11).

$$\begin{aligned}\frac{d[G_{i/o}]}{dt} &= [P2Y12]_{act}k_{f,2} - k_{deg,1}[G_{i/o}]^2 \\ \frac{d[PI3K]_s}{dt} &= k_{f,3}Signal_{Ca^{2+}}([PI3K]_{tot} - [PI3K]_s - [PI3K]_{act}) \\ &\quad - k_{b,3}[PI3K]_s - k_{f,4}[PI3K]_s[G_{i/o}] \\ \frac{d[PI3K]_{act}}{dt} &= k_{f,4}[PI3K]_s[G_{i/o}] - k_{b,4}[PI3K]_{act} \\ \frac{d[pAkt]}{dt} &= k_{f,5}([Akt]_{total} - [pAkt])[PI3K]_{act} - k_{b,5}[pAkt]\end{aligned} \tag{S11}$$

Chemotaxis in our model scales with the active states of pAkt[22] and CaM[8] :

$$\begin{aligned}Signal_{pAkt} &= \frac{1}{1 + \left(\frac{kd_{pAkt}}{[pAkt]}\right)} \\ Signal_{CaM} &= \frac{1}{1 + \left(\frac{kd_{CaM}}{[Ca_4CaM] - [CaM]_0}\right)} \\ \frac{dV_{mig}}{dt} &= k_{f,mig}(Signal_{pAkt} + 0.05Signal_{CaM}) - k_{r,mig}V_{mig} \\ \Delta Distance_{mig} &= V_{mig}\Delta t\end{aligned} \tag{S12}$$

Table S15: Parameters for simulating $P2Y12$-mediated Chemotaxis listed in Eqn S10-S12

Parameters	Values	Units
f_{P2Y12}	0.7	
$f_{Ca^{2+}}$	0.3	
$k_{f,1}$	0.008	1/s
$k_{b,1}$	0.02	1/s
$k_{f,2}$	0.1	1/s
$k_{b,2}$	0.01	1/s
$k_{f,3}$	0.01	1/s
$k_{b,3}$	0.01	1/s
$k_{f,4}$	0.00001	1/s
$k_{b,4}$	0.01	1/s
$k_{f,5}$	0.001	1/s
$k_{b,5}$	0.1	1/s
$k_{f,6}$	0.001	1/s
$k_{b,6}$	0.01	1/s
$k_{deg,1}$	0.05	1/s
$[P2Y12]_{total}$	100	
$[PI3K]_{total}$	100	
$[Akt]_{total}$	100	

S.2.7 Degradation of ATP by NTPDase1

Ectonucleoside triphosphate diphosphohydrolase-1 (E-NTPDase), also known as CD39, is a plasma-membrane protein that plays an important role in microglial migration by balancing ATP and adenosine molecules [9]. We have adapted the kinetic model of ATP degradation into ADP and AMP by CD39 introduced in the [18] to determine the ATP and ADP available for the purinergic receptors in our microglial models:

$$\begin{aligned} \frac{d[ATP]}{dt} &= -k_{1,deg}[ATP] \\ \frac{d[ADP]}{dt} &= k_{1,deg}[ATP] - k_{2,deg}[ADP] \\ \frac{d[AMP]}{dt} &= k_{2,deg}[ADP] \end{aligned} \tag{S13}$$

The parameters for these equations controlling nucleotide availability are given in Table S16.

Table S16: Parameters for the degradation of ATP by NTPDase listed in S13

Parameters	Values	Units
$k_{1,deg}$	0.002	1/s
$k_{2,deg}$	0.008	1/s

S.3 Figures

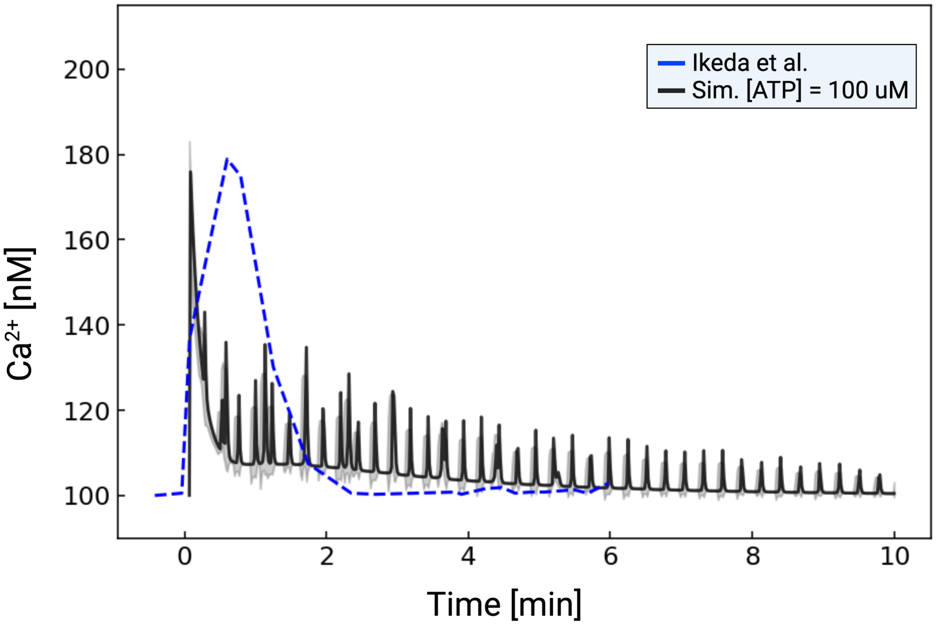

Figure S1: Predicted (black) versus experimentally measured (blue) Ca^{2+} responses following 100 μM ATP. [14] .

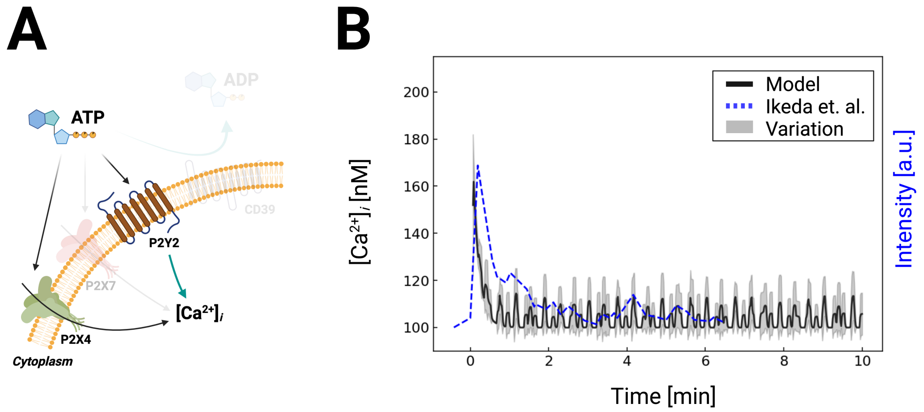

Figure S2: A) Schematic for Ca^{2+} waveforms generated by $P2X4$ and $P2Y2$ in response to 1mM ATP for 10 minutes, which does not include the degradation of ATP by CD39. B) Comparison of predicted (black: moving average with a window size of 8 reported along the left y-axis.) and experimentally-measured [14] (blue-dashed) Ca^{2+} transients reported along the right y-axis .

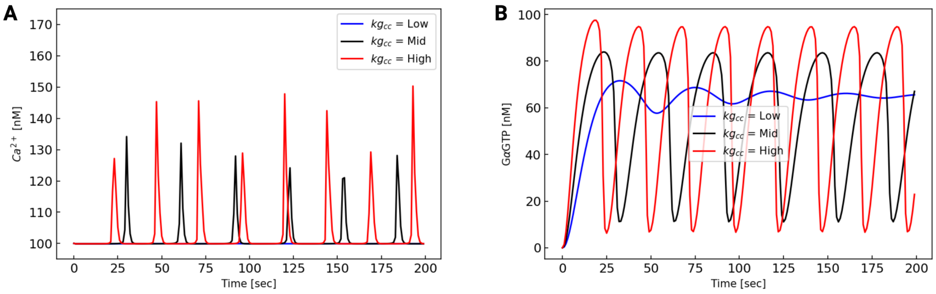

Figure S3: A) Intracellular Ca^{2+} transients and their corresponding B) oscillation of active $G_{\alpha q}$ with respect to $k_{g,cc}$ that controls the activation of $G_{\alpha q}$.

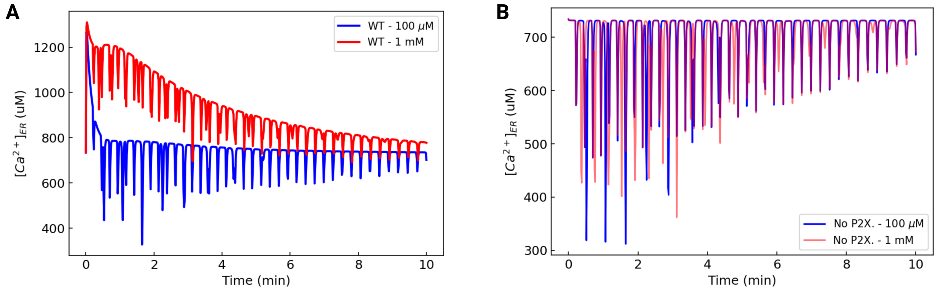

Figure S4: Comparison between Ca^{2+} transients induced by ER Ca^{2+} release via IP_3-mediated pathway at 100 µM and 1 mM ATP concentrations in cytoplasm (A) and ER lumen (B). The WT microglia model was used for this prediction. The faded lines denote the contribution by $P2Y$ receptor activation that results in ER Ca^{2+} release to the cytosolic domain. The data demonstrate the relationship between cytosolic and ER Ca^{2+} transients, which suggest that roughly 43.7% of Ca^{2+} is drawn from the ER at low ATP vs. 33.3% at high ATP.

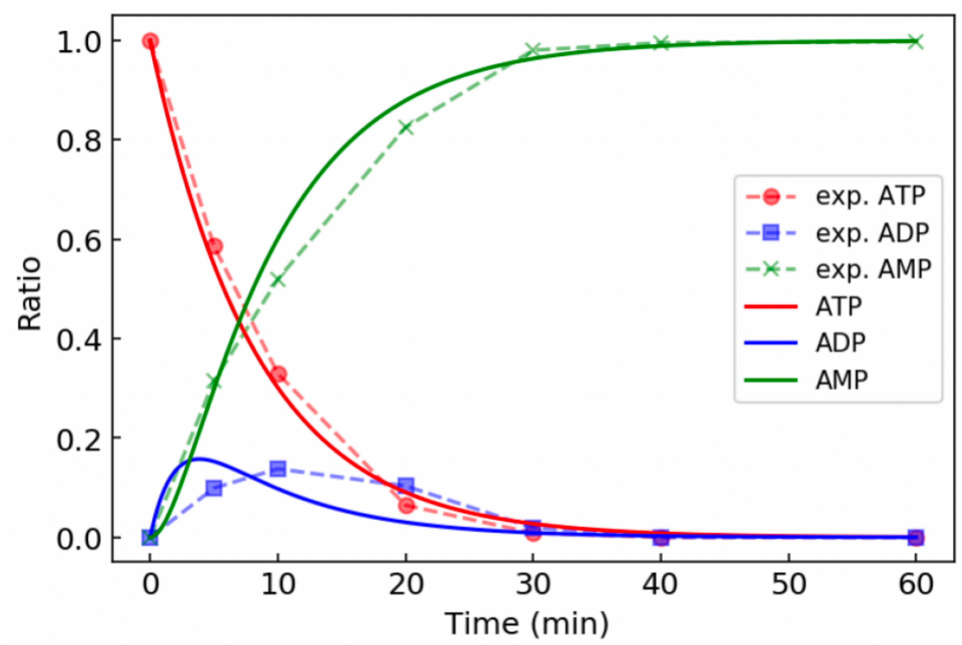

Figure S5: Validations of our model of ATP/ADP hydrolysis into AMP by CD39 against experimental data (dashed). Each nucleotide concentration was measured by Kukulski *et al* [18] in COS-7 cells.

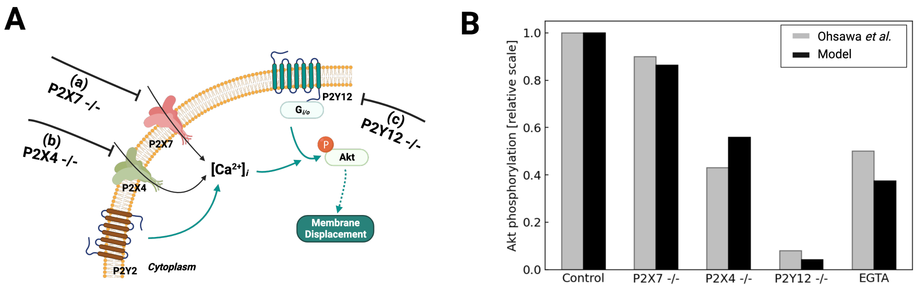

Figure S6: A) Schematic of P2Y12- and Ca-mediated migration in response to ATP, assuming control, P2X7 -/- (a), P2X4 -/- (b) and P2Y12 -/- (c) conditions. B) Predicted pAkt levels (black) versus experimental measurements by Ohsawa *et al* under control and a-c conditions [14].

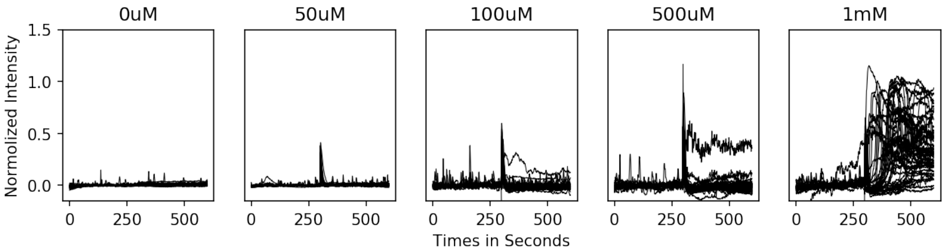

Figure S7: Traces of the fluorescence to measure the ATP-mediated Ca^{2+} transients in BV2 cells.

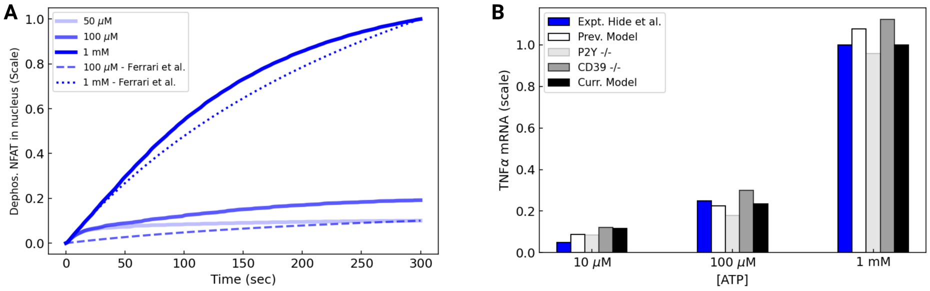

Figure S8: Predictions of dephosphorylated NFAT in nucleus over time (A) and TNFα mRNA with various computation configurations and comparison to the previously developed model[4] with respect to amplitude of stimulation (5). All simulations in B) were performed for 5 minutes. The plot is in the unit of scale, whose basis is the maximum increment measured by the current model.

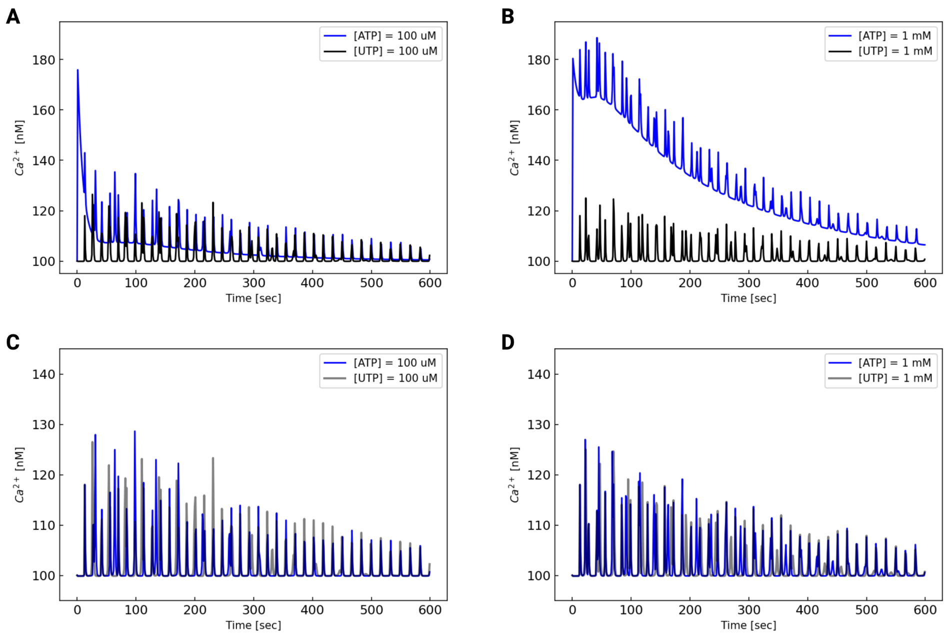

Figure S9: Simulation of Ca^{2+} transients in BV2 with 100 µM and 1 mM ATP and UTP. Primary microglia with low (A) and high (B) ATP/UTP concentration whereas BV2 cells with low (C) and high (D) concentration of the stimulant. In this case, UTP selectively actives only P2Y2 receptors. [27].

Bibliography

[1] Barbera-Cremades, M., Gomez, A. I., Baroja-Mazo, A., Martinez-Alarcon, L., Martinez, C. M., de Torre-Minguela, C., and Pelegrin, P. P2x7 receptor induces tumor necrosis factor alpha converting enzyme activation and release to boost tnfa production. *Frontiers in immunology 8* (2017), 862.

[2] Bazzazi, H., Sang, L., Dick, I. E., Joshi-Mukherjee, R., Yang, W., and Yue, D. T. Novel fluorescence resonance energy transfer-based reporter reveals differential calcineurin activation in neonatal and adult cardiomyocytes. *Journal of Physiology 593*, 17 (2015), 3865--3884.

[3] Chessell, I. P., Michel, A. D., and Humphrey, P. P. A. Properties of the pore-forming P2X7 purinoceptor in mouse NTW8 microglial cells. *British Journal of Pharmacology 121*, 7 (1997), 1429--1437.

[4] Chun, B. J., Stewart, B. D., Vaughan, D. D., Bachstetter, A. D., and Kekenes-Huskey, P. M. Simulation of p2x-mediated calcium signalling in microglia. *The Journal of Physiology 597*, 3 (2019), 799--818.

[5] Cooling, M. T., Hunter, P., and Crampin, E. J. Sensitivity of NFAT cycling to cytosolic calcium concentration: Implications for hypertrophic signals in cardiac myocytes. *Biophysical Journal 96*, 6 (2009), 2095--2104.

[6] Cuthbertson, K., and Chay, T. Modelling receptor-controlled intracellular calcium oscillators. *Cell calcium 12*, 2-3 (1991), 97--109.

[7] De Young, G. W., and Keizer, J. A single-pool inositol 1, 4, 5-trisphosphate-receptor-based model for agonist-stimulated oscillations in ca2+ concentration. *Proceedings of the National Academy of Sciences 89*, 20 (1992), 9895--9899.

[8] Fan, Y., Xie, L., and Chung, C. Y. Signaling pathways controlling microglia chemotaxis. *Molecules and cells 40*, 3 (2017), 163.

[9] Färber, K., Markworth, S., Pannasch, U., Nolte, C., Prinz, V., Kronenberg, G., Gertz, K., Endres, M., Bechmann, I., Enjyoji, K., Robson, S. C., and Kettenmann, H. The ectonucleotidase cd39/entpdase1 modulates purinergic-mediated microglial migration. *Glia 56*, 3 (2008), 331--341.

[10] Garcia-Guzman, M., Soto, F., Gomez-Hernandez, J. M., Lund, P. E., and Stuehmer, W. Characterization of recombinant human P2X4 receptor reveals pharmacological differences to the rat homologue. *Molecular pharmacology 51*, 1 (1997), 109--118.

[11] Heineke, J., and Molkentin, J. D. Regulation of cardiac hypertrophy by intracellular signalling pathways. *Nature Reviews Molecular Cell Biology 7*, 8 (2006), 589--600.

[12] Hickman, S. E., Kingery, N. D., Ohsumi, T. K., Borowsky, M. L., Wang, L.-c., Means, T. K., and El Khoury, J. The microglial sensome revealed by direct rna sequencing. *Nature neuroscience 16*, 12 (2013), 1896.

[13] Hide, I., Tanaka, M., Inoue, A., Nakajima, K., Kohsaka, S., Inoue, K., and Nakata, Y. Extracellular ATP triggers tumor necrosis factor-a release from rat microglia. *Journal of Neurochemistry 75*, 3 (jan 2000), 965--972.

[14] Ikeda, M., Tsuno, S., Sugiyama, T., Hashimoto, A., Yamoto, K., Takeuchi, K., Kishi, H., Mizuguchi, H., Kohsaka, S.-i., and Yoshioka, T. Ca2+ spiking activity caused by the activation of store-operated ca2+ channels mediates tnfa release from microglial cells under chronic purinergic stimulation. *Biochimica et Biophysica Acta (BBA)-Molecular Cell Research 1833*, 12 (2013), 2573--2585.

[15] Kettenmann, H., Hanisch, U.-K., Noda, M., and Verkhratsky, A. Physiology of Microglia. *Physiol Rev 91* (2011), 461--553.

[16] Khadra, A., Yan, Z., Coddou, C., Tomić, M., Sherman, A., and Stojilkovic, S. S. Gating properties of the P2X2a and P2X2b receptor channels: Experiments and mathematical modeling. *The Journal of general physiology 139*, 5 (2012), 333--348.

[17] Khadra, A., Yan, Z., Coddou, C., Tomic, M., Sherman, A., and Stojilkovic, S. S. Gating properties of the P2X2a and P2X2b receptor channels: Experiments and mathematical modeling. *The Journal of general physiology 139*, 5 (2012), 333--348.

[18] Kukulski, F., Levesque, S., Lavoie, E., Lecka, J., Bigonnesse, F., Knowles, A., Robson, S., Kirley, T., and Sevigny, J. Comparative hydrolysis of p2 receptor agonists by ntpdases 1, 2, 3 and 8. *Purinergic signalling 1*, 2 (2005), 193.

[19] Litosch, I. G protein co-signaling and challenges for translational research. *Translational Neuroscience 4*, 1 (2013), 66--73.

[20] Nagamoto-Combs, K., and Combs, C. K. Microglial Phenotype Is Regulated by Activity of the Transcription Factor, NFAT (Nuclear Factor of Activated T Cells). *Journal of Neuroscience 30*, 28 (jul 2010), 9641--9646.

[21] Noda, Mami and Verkhratsky, A. Physiology of Microglia. In *Neuroglia*, B. Kettenmann, Helmut and Ransom, Ed., 3 ed. Oxford Univ Press, 2012, pp. 223--237.

[22] Ohsawa, K., Imai, Y., Sasaki, Y., and Kohsaka, S. Microglia/macrophage-specific protein Iba1 binds to fimbrin and enhances its actin-bundling activity. *Journal of Neurochemistry 88*, 4 (jan 2004), 844--856.

[23] Ohsawa, K., Irino, Y., Nakamura, Y., Akazawa, C., Inoue, K., and Kohsaka, S. Involvement of p2x4 and p2y12 receptors in atp-induced microglial chemotaxis. *Glia 55*, 6 (2007), 604--616.

[24] Robson, S. C., Sevigny, J., and Zimmermann, H. The e-ntpdase family of ectonucleotidases: structure function relationships and pathophysiological significance. *Purinergic signalling 2*, 2 (2006), 409.

[25] Shannon, T. R., Wang, F., Puglisi, J., Weber, C., and Bers, D. M. A mathematical treatment of integrated Ca dynamics within the ventricular myocyte. *Biophysical Journal 87*, 5 (2004), 3351--3371.

[26] Skupin, A., Kettenmann, H., Winkler, U., Wartenberg, M., Sauer, H., Tovey, S. C., Taylor, C. W., and Falcke, M. How does intracellular Ca2+ oscillate: by chance or by the clock? *Biophysical journal 94*, 6 (mar 2008), 2404--11.

[27] Sophocleous, A., R., Miles, A., N., Ooi, L., Sluyter, ., and R. P2y2 and p2x4 receptors mediate ca2+ mobilization in dh82 canine macrophage cells. *International Journal of Molecular Sciences 21*, 22 (2020), 8572.

[28] Toulme, E., and Khakh, B. S. Imaging P2X4 receptor lateral mobility in microglia: Regulation by calcium and p38 MAPK. *Journal of Biological Chemistry 287*, 18 (2012), 14734--14748.

[29] Trang, T., Beggs, S., Wan, X., and Salter, M. W. P2X4-Receptor-Mediated Synthesis and Release of Brain-Derived Neurotrophic Factor in Microglia Is Dependent on Calcium and p38-Mitogen-Activated Protein Kinase Activation. *Journal of Neuroscience 29*, 11 (2009), 3518--3528.

[30] Umpierre, A. D., Bystrom, L. L., Ying, Y., Liu, Y. U., Worrell, G., and Wu, L.-J. Microglial calcium signaling is attuned to neuronal activity in awake mice. *Elife 9* (2020), e56502.

[31] Young, P., Ferguson, C., Bañuelos, S., and Gautel, M. Molecular structure of the sarcomeric Z-disk: two types of titin interactions lead to an asymmetrical sorting of α-actinin. *The EMBO journal 17*, 6 (1998), 1614--1624.

[32] Zawadzka, M., Dabrowski, M., Gozdz, A., Szadujkis, B., Sliwa, M., Lipko, M., and Kaminska, B. Early steps of microglial activation are directly affected by neuroprotectant FK506 in both in vitro inflammation and in rat model of stroke. *Journal of Molecular Medicine 90*, 12 (dec 2012), 1459--1471.

[33] Zawadzka, M., Dabrowski, M., Gozdz, A., Szadujkis, B., Sliwa, M., Lipko, M., and Kaminska, B. Early steps of microglial activation are directly affected by neuroprotectant FK506 in both in vitro inflammation and in rat model of stroke. *Journal of Molecular Medicine 90*, 12 (dec 2012), 1459--1471.
